# Corilagin Ameliorates Atherosclerosis in Peripheral Artery Disease via the Toll-Like Receptor-4 Signaling Pathway *in vitro* and *in vivo*

**DOI:** 10.3389/fimmu.2020.01611

**Published:** 2020-08-06

**Authors:** Yiqing Li, Yujie Wang, Yunfei Chen, Yao Wang, Shaojun Zhang, Pan Liu, Zhilin Chen, Peng Song, Lei Luo, Yingying Luo, Yiping Dang, Lei Zhao

**Affiliations:** ^1^Department of Vascular Surgery, Union Hospital, Tongji Medical College, Huazhong University of Science and Technology, Wuhan, China; ^2^Department of Infectious Diseases, Renmin Hospital of Wuhan University, Wuhan, China; ^3^National & Local Joint Engineering Research Center for High-Throughput Drug Screening Technology, State Key Laboratory of Biocatalysis and Enzyme Engineering, Hubei University, Wuhan, China; ^4^Department of Pediatrics, Wuchang Hospital, Wuhan, China; ^5^Department of Infectious Diseases, Dongxihu People's Hospital, Wuhan, China; ^6^Department of Breast and Thyroid Surgery, People's Hospital of Ningxia Hui Autonomous Region, Yinchuan, China; ^7^Department of Gastroenterology, The Second People's Hospital of China Three Gorges University, Yichang, China; ^8^School of Clinical Medical, Hubei University of Chinese Medicine, Wuhan, China; ^9^Department of Infectious Diseases, Union Hospital, Tongji Medical College, Huazhong University of Science and Technology, Wuhan, China

**Keywords:** corilagin, peripheral artery disease, atherosclerosis, toll-like receptor 4, monocyte/macrophage

## Abstract

We investigated if corilagin can ameliorate or reverse atherosclerotic development via the toll-like receptor 4 (TLR4) signaling pathway *in vitro* and *in vivo*. Ana-1 cells or mouse peritoneal macrophages (MPMs) were stimulated with oxidized low-density lipoprotein followed by corilagin treatment. TLR4 expression in Ana-1 cells was upregulated by lentiviral transduction and downregulated by small interfering RNA. Peripheral blood mononuclear cells (PBMCs), plasma samples, and femoral arteries were collected from rats exhibiting peripheral artery disease (PAD). mRNA and protein expression of TLR4 and downstream molecules were decreased significantly by corilagin treatment in Ana-1 cells, MPMs, and rat PBMCs, and the reduction remained irrespective of downregulation or upregulation of TLR4 expression in Ana-1 cells. Corilagin also exerted a prominent effect on changes in plasma levels of cytokines and the pathologic manifestation of atherosclerosis in femoral arteries. Corilagin could ameliorate the development of atherosclerotic plaques by inhibiting the TLR4 signaling pathway in monocyte/macrophages and reduce the release of proinflammatory cytokines. This study provides a new therapeutic target and new *niche* targeting drug to oppose atherosclerosis and reveals the enormous potential of corilagin for control of PAD in humans.

## Introduction

Due to an increase in the average age of the world population, peripheral artery disease (PAD) is the third-leading cause of atherosclerotic cardiovascular morbidity. PAD was reported to affect 202 million people worldwide in 2010, and PAD prevalence has increased to 28.7% in low-income or middle-income countries, and 13.1% in high-income countries (1, 2).

As one of the most common types of PAD, arteriosclerosis obliterans is characterized by pathologic changes of atherosclerosis in peripheral arteries with a series of ischemic symptoms (3). Atherosclerosis has been studied for a long time, and inflammation has been recognized as a major and independent factor in atherogenesis in recent years (4). However, the molecular and cellular pathways of atherosclerosis have not been clarified entirely.

The cells involved in the innate immune response (especially mononuclear macrophages) promote atherogenesis and regulate the stability of plaques in pivotal ways, such as participating in foam-cell formation, mediating the release and effects of cytokines, as well as interacting with endothelial cells and smooth muscle cells (5, 6). Toll-like receptors (TLRs) are vital components of the innate immune system known for their ability to induce the inflammatory response at the outset (7). A more important fact is that TLRs are considered to be associated with atherosclerosis through management of expression of some major molecules (8).

TLR4 is one of the most-reported critical promotors in various processes of atherosclerosis. TLR4 plays a vital part in coronary arteriosclerosis disease and acute myocardial infarction due to its increased expression/activation in peripheral blood mononuclear cells (PBMCs) (9, 10). As an activator of a series of inflammatory cascades in a nuclear factor-kappa B (NK-κB)-dependent fashion (11), TLR4 overexpression can lead to excess release of proinflammatory cytokines and chemokines, such as tumor necrosis factor (TNF)-α, interleukin (IL)-1, IL-6, and type-I interferon (IFN)-α (9). Thus, finding new therapeutic agents for atherosclerosis by regulating the TLR4 signaling pathway is a rational approach.

Corilagin is a ^1^C_4_/B-ellagitannin (12) isolated from *Phyllanthus urinaria*. Corilagin can protect against herpes simplex virus-1-induced encephalitis by reducing expression of TNF-α and NF-κB through inhibition of the TLR2 signaling pathway (13). Corilagin can suppress the interleukin-13/Janus kinase/signal transducer and activator of transcription-6 and microRNA-21/smad7/extracellular signal-regulated kinase signaling pathways in macrophages to reduce fibrosis severity (14). Corilagin has anti-oxidative and anti-inflammation function due to its suppression of expression of proinflammatory cytokines and mediators such as TNF-α, IL-6, inducible nitric oxide synthase, and cyclo-oxygenase-2 (15). The pharmacodynamic effects of corilagin (anti-hyperalgesia, anti-hypertensive, thrombolytic, and anti-cancer) have also been documented (16–18). However, whether corilagin could treat atherosclerosis is still unknown.

In this study, we used the Ana-1 cell line, primary abdominal macrophages of mice, and a PAD model in rats to explore the effect of corilagin on amelioration of atherosclerosis development via the TLR4 signaling pathway. In this way, we wished to provide a new strategy to prevent and treat atherosclerosis in humans.

## Materials and Methods

### Study Design

Oxidized low-density lipoprotein (Ox-LDL) is an endogenous ligand for TLR4 in atherosclerosis and can also upregulate TLR4 expression (19, 20). We used ox-LDL *in vitro* to stimulate Ana-1 cells to establish a cell model of atherosclerosis. In the *in vivo* study, rats were fed a high-fat and high-cholesterol diet for 3 months after the intima of the femoral artery had been destroyed mechanically. Corilagin and aspirin were used after completing model creation. We measured expression of the molecules involved in the TLR4 signaling way [TLR4, toll-interleukin 1 receptor domain-containing adaptor protein (TIRAP), myeloid differentiation factor (MyD)88, tumor necrosis factor receptor-associated factor (TRAF)6, nuclear factor-kappa B essential modulator (NEMO), mitogen-activated protein kinase p38 (p38), and interferon regulatory factor 5 (IRF5)] to discover the targets of their anti-atherosclerosis effects *in vitro* and *in vivo*. The extent of femoral-artery atherosclerosis was observed to estimate the efficacy of drugs. Based on our data, we aimed to find a new therapeutic method to treat atherogenesis, against which existing drugs have little effect.

### Ethical Approval of the Study Protocol

Animal experiments and procedures followed the *Guidelines for the Care and Use of Laboratory Animals* (National Institutes of Health, Bethesda, MD, USA). The study protocol was approved by the Animal Care and Use Committee of Tongji Medical College [[2017] IACUC number: 834] within Huazhong University of Science and Technology (Wuhan, China).

### Reagents

Corilagin standard substance (purity > 99%) for cells was purchased from the China National Institute for the Control of Pharmaceutical and Biological Products. Corilagin for animal experimentation was extracted and provided by Professor Rong-Zeng Huang (School of Pharmacy, Hubei University of Chinese Medicine). Cell Counting Kit (CCK)-8 was purchased from Dojindo Molecular Technologies (Kumamoto, Japan). Ox-LDL was oxidized from human LDL and analyzed by electrophoresis on agarose gel.

### Cell Line and Administration

Ana-1 is a well-established murine macrophage cell line. It was obtained from the Type Culture Collection of the Chinese Academy of Sciences (Shanghai, China). Cells were cultured in RPMI-1640 medium containing 10% fetal bovine serum (FBS) in an incubator at 37°C in an atmosphere of 5% CO_2_ and saturated humidity (21).

### CCK8 Assay for Measuring the Toxicity of Corilagin on Ana-1 Cells

We used the CCK8 assay to evaluate the cytotoxicity of corilagin. Ana-1 cells were inoculated in 96-well plates (10^4^/ml) with corilagin (12.5–800 μg/ml). RPMI-1640 with and without cells was set as the positive control and negative control, respectively. After 24 h, cell morphology was observed under a microscope (IMT-2, Olympus, Tokyo, Japan). Then, 10 μl of CCK8 was added to each well for 2-h incubation before measuring absorbance at 450 nm using a microplate reader (DG-235, Thermo Fisher Scientific, USA).

### Determination of Reaction Conditions for Ox-LDL

According to previous studies (20, 22, 23), we stimulated Ana-1 cells with ox-LDL (12.5–200 μg/ml). Then, we determined the appropriate concentration on the basis of mRNA expression of some molecules involved in the TLR4 signaling pathway by quantitative real-time polymerase chain reaction (qRT-PCR). Similarly, we stimulated Ana-1 cells with ox-LDL for 6–48 h to ascertain the appropriate incubation time.

### Transfection of Small Interfering (si)RNA in Ana-1 Cells

TLR4 expression was downregulated by four sets of siRNA transfection. siRNA was purchased from Ribobio (Guangzhou, China) and the sequences were shown in Table 1. We added siRNA (100 pmol) to 5 μl of Lipofectamine™ 2000 (Invitrogen, Carlsbad, CA, USA), FBS, and RPMI-1640 into six-well plates to ensure that the final concentration of siRNA was 0.02 nmol/μl. Six hours later, we incubated cells with FBS and RPMI-1640 for 48 h. The efficiency of TLR4 downregulation was checked by qRT-PCR and Western blotting. Although all the four sets of siRNA could reduce the expression of TLR4, effect of siRNA-3 was the most significant (Supplemental Figure 1). Therefore, cells transfected by siRNA-3 were treated with other reagents for further experimentation.

**Table 1:** siRNA sequences used in this study.

	**Sense**	**Antisense**
siRNA-1	GAAAUGAGCUGGUAAAGAATT	UUCUUUACCAGCUCAUUUCTT
siRNA-2	GCAUAGAGGUAGUUCCUAATT	UUAGGAACUACCUCUAUGCTT
siRNA-3	GAACAAAUGACAUGUGCAATT	UUGCACAUGUCAUUUGUUCTT
siRNA-4	GGGAGAAUUUAAAGAUGAATT	UUCAUCUUUAAAUUCUCCCTT

### TLR4 Overexpression in Ana-1 Cells via the Lentiviral Vector

TLR4 expression was upregulated by lentivirus transfection. The lentiviral vector contained a pre-TLR4 sequence, enhanced green fluorescent protein (GFP) and resistance to puromycin (PuroR). First, we transfected Ana-1 cells in the enhanced infection solution with lentivirus at a multiplicity of infection of 40 for 72 h. Then, we screened out untransfected cells with puromycin (Sigma–Aldrich, St. Louis, MO, USA) for 1 week until all cells expressed green fluorescence as observed under an IX2-series fluorescence microscope (Olympus, Tokyo, Japan). We cultured the remainder of cells for further experimentation. The transfection efficiency was measured by qRT-PCR and Western blotting.

### Comparing Aspirin With TAK-242 in Ana-1 Cells After Ox-LDL Stimulation

We treated ox-LDL-stimulated Ana-1 cells with corilagin, aspirin, and TAK-242 (TLR4 neutralizing antibodies, ApexBio, Houston, TX, USA) to compare the effects of the reduction of TLR4 expression. The results are shown in Supplemental Figure 2. We found that TAK-242 could not effectively reduce the mRNA or protein expression of TLR4 and MyD88 compared to ox-LDL group. The reduction in the aspirin group was relatively significant.

In previous studies, researchers determined that TAK-242 inhibited TLR4 signal pathway by binding to TLR4 intracellular domain and interfering the interactions between TLR4 and adaptor molecules (24, 25). In contrast, aspirin was found that could reduce the expression of TLR4 in C26 cells and hepatic stellate cells (26, 27). Our results were consistent with these studies. Considering that aspirin has already been widely applied in the clinical field and its pharmacological effect on TLR4, we chose aspirin as the positive control in our further experiments.

### Corilagin Treatment for Ana-1 Cells After Ox-LDL Stimulation

Ana-1 cells were divided into groups: untreated, model, high concentration (100 μg/ml), medium concentration (50 μg/ml), low concentration (25 μg/ml), and positive control (aspirin). Except for the untreated group, the other groups were stimulated with ox-LDL (25 μg/ml) for 24 h, followed by no treatment, or treatment with corilagin at low, medium, and high concentrations, or treatment with aspirin.

### Mouse Peritoneal Macrophages (MPMs) Extraction

C57BL/6 mice were purchased from HFK Bios (Beijing, China). Next, 2 ml of 4% mercaptoacetate broth medium was injected into the abdominal cavity. Three days later, mice were injected (i.p.) with 5 ml of phosphate-buffered saline (PBS) containing 3% FBS. Five minutes later, mice were killed, and peritoneal fluid was removed and centrifuged at 250 *g* for 8 min at room temperature. The cell pellet was resuspended in RPMI-1640 with 10% FBS and incubated in six-well plates at 37°C in an atmosphere of 5% CO_2_ for 2 h. Adherent cells were cultured and used for further experimentation.

### Creation of an Animal Model of Atherosclerosis

Sprague–Dawley rats were purchased from the Experimental Animal Center of Tongji Medical College. Forty-two male Sprague–Dawley rats (3 weeks, 100–150 g) were divided randomly into seven groups. Corilagin groups, the aspirin group, and the model group were fed a high-fat and high-cholesterol diet (D12109C; HFK Bios) for 3 months after mechanical injury to the intima of the femoral artery. Briefly, we separated a segment of the femoral artery and clamped both ends temporarily, and filled it with sterile water to cause extracellular hypoosmosis that would lead to rupture of endothelial cells. This method is an improvement on guidewire-induced injury method that was mentioned in published articles (28, 29). According to our previous study (30), we designed a preliminary experiment to verify the concentrations and mortality curve (Supplemental Figure 3). Then, we treated rats with high (40 mg/kg·day), medium (20 mg/kg·day), or low (10 mg/kg·day) concentrations of corilagin in corilagin groups, aspirin (20 mg/kg·day) in the aspirin group, or physiologic (0.9%) saline in the control group, sham-operation group, and model group, by means of intragastric administration for 1 month. The control group and sham-operated group were fed a normal diet. Finally, we separated and extracted PBMCs by density gradient centrifugation for further experimentation.

### Flow Cytometry (FCM) for Measuring TLR4 Expression

PBMCs were resuspended using PBS. Then, they were reacted with fluorescein isothiocyanate anti-cluster of differentiation (CD) 45 antibody (catalog number, 202205; BioLegend, San Diego, CA, USA), phycoerythrin anti-CD86 antibody (200308; BioLegend), and allophycocyanin anti-TLR4 antibody (145405; BioLegend). After washing twice with PBS, PBMCs were analyzed on a FACSCalibur™ flow cytometer (BD Biosciences, San Jose, CA, USA).

### Analyses of Femoral-Artery Plaques

We wished to visualize the area of plaques. Femoral arteries were separated and cut in frozen-tissue sections of thickness 5–10 μm. They were stained with 0.5% Oil Red O solution in isopropanol for 10 min and washed in water and 60% isopropanol. Stained areas were quantified using ImageJ (National Institutes of Health).

### Enzyme-Linked Immunosorbent Assays (ELISAs)

The level of IL-1, IL-6, IFN, and TNF-α in the plasma of rats was measured by ELISAs. IL-1 (E-EL-R0012c), IL-6 (E-EL-R0015c), IFN (E-EL-R0009c), and TNF-α (E-EL-R0019c) ELISA kits were purchased from Elabscience (Wuhan, China). The procedures were conducted according to the instruction manuals for the kits. Sterile PBS was used as the control.

### qRT-PCR for Measuring mRNA Expression

mRNA expression of TLR4, TIRAP, MyD88, TRAF6, NEMO, p38, and IRF5 was measured by qRT-PCR. The operation and analyses were undertaken according to our previously published protocol (31). Total RNA was isolated from Ana-1 cells, MPMs, or rat PBMCs using RNAiso Plus (TaKaRa Biotechnology, Dalian, China). Total RNA was reversed to complimentary (c)DNA following the protocol of the PrimeScript™ RT Reagent kit (TaKaRa Biotechnology). Real-time PCRs were carried out according to the instructions for the SYBR™ Premix Ex Taq kit (TaKaRa Biotechnology) at 95°C for 30 s, 95°C for 5 s and 60°C for 30 s, for 40 cycles, and 95°C for 15 s, 60°C for 1 min, and 95°C for 15 s using a StepOne™ Plus device (Applied Biosystems, Foster City, CA, USA). Data were analyzed using the 2^−ΔΔCT^ method. Glyceraldehyde 3-phosphate dehydrogenase was used as the reference gene. The primers for qRT- PCR are shown in Table 2, and their specificity was checked by Primer BLAST.

**Table 2:** Primer sequences used in this study.

		**Mouse**	**Rat**
TLR-4	Forward	TCTGGGGAGGCACATCTTCT	CTCACAACTTCAGTGGCTGGATTTA
	Reverse	AGGTCCAAGTTGCCGTTTCT	TGTCTCCACAGCCACCAGATTC
TIRAP	Forward	CCAAGAAGCCTCGAGACAAG	GCCCATTCATCAATAATCAC
	Reverse	GTGGCGAGGTAGGTGACATT	ATCCATCCATCCACCCACTC
MyD88	Forward	CATACCCTTGGTCGCGCTTA	AGGAGATGGGTTGTTTCGAGTAC
	Reverse	CCAGGCATCCAACAAACTGC	CTCACGGGTCTAACAAGGCTA
TRAF6	Forward	AAACCACGAAGAGGTCATGG	GTCTGAAGCAGTAATAAGGCAAAAC
	Reverse	GCGGGTAGAGACTTCACAGC	CCCCATGTCAAAGCGGGTAG
p38	Forward	ATCATTCACGCCAAAAGGAC	ATGTCTCAGGAGAGGCCCACGTTCT
	Reverse	AGCTTCTGGCACTTCACGAT	TCAGGAGTCCATTTCTTCTTGGTC
NEMO	Forward	GGTGGAGAGACTGAGCTTGG	GGCAGCACTCCTTATCAA
	Reverse	CTAAAGCTTGCCGATCCTTG	GGTGTCGTCCCATCGTAG
IRF5	Forward	AATACCCCACCACCTTTTGA	TGTAGTAGCCACAACCCAAAAGA
	Reverse	TTGAGATCCGGGTTTGAGAT	GGGGGAGTCAGAGACAGAGAAA
GAPDH	Forward	CAGCAAGGACACTGAGCAAGA	ACGGCACAGTCAAGGCTGAGA
	Reverse	GCCCCTCCTGTTATTATGGGG	CGCTCCTGGAAGATGGTGAT

### Western Blotting for Measuring Protein Expression

Protein expression of TLR4, TIRAP, MyD88, TRAF6, NEMO, p38, and IRF5 was measured by Western blotting, as described previously by our research team (32). Protein samples isolated from Ana-1 cells, MPMs, or rat PBMCs were separated by sodium dodecyl sulfate–polyacrylamide gel electrophoresis (8–12%), and transferred to polyvinylidene difluoride (PVDF) membranes following blockade with 5% non-fat milk in Tris-buffered saline and Tween 20 (TBST) buffer for 1 h at room temperature. Then, PVDF membranes were incubated with primary antibodies overnight at 4°C, washed thrice with TBST, and incubated with horseradish peroxidase-labeled goat anti-rabbit or goat anti-mouse antibodies for 1 h at room temperature. The dilution of the primary antibodies was 1:1,000. Rabbit anti-mouse MyD88 and TIRAP were obtained from ABclonal (Beijing, China). Rabbit anti-mouse TLR4, TRAF6, NEMO, p38MAPK, and IRF5 were obtained from Proteintech (Beijing, China).

### Statistical Analyses

Data were analyzed using SPSS v24.0 (IBM, Armonk, NY, USA). Data are the mean ± SD. The significance of differences among groups was determined by one-way analysis of variance (ANOVA), followed by the Student–Newman–Keuls (S–N–K) *q* test. *P* < 0.05 was considered significant.

## Results

### Cytotoxicity and the Effect on the TLR4 Signaling Pathway of Corilagin in Ana-1 Cells

**Figure 1:**
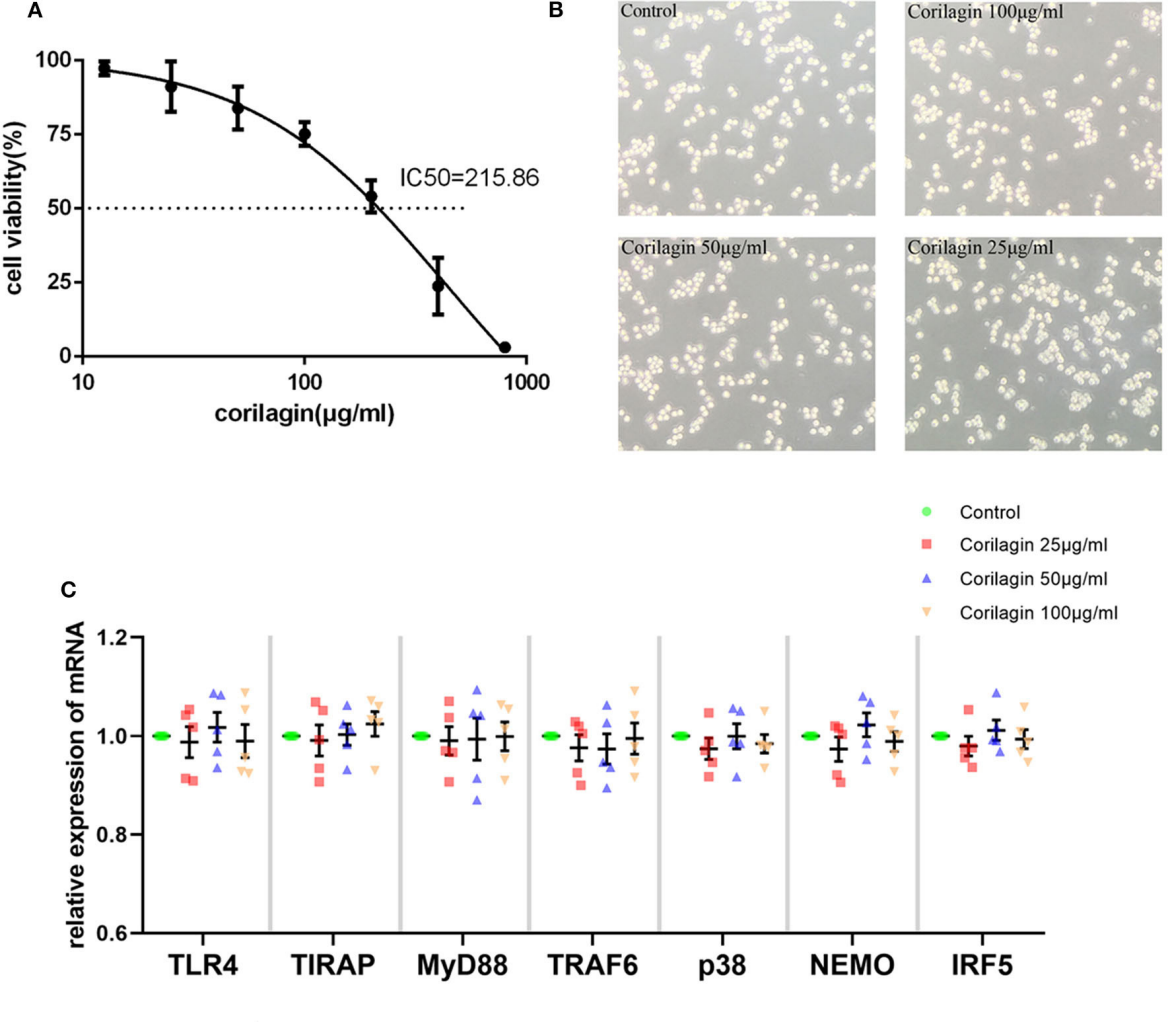
Cytotoxicity of Corilagin **(A)** CCK8 assay of Ana-1 cell viability. The concentration–effect curve was *Y* = (152.95/(1+10^logX−2.635^)−51.95. IC_50_ = 215.86 μg/ml. **(B)** Morphology of Ana-1 cells after corilagin treatment for 24 h. **(C)** mRNA expression measured by qRT-PCR. Data are the mean and SEM. No significant difference was found between groups determined by one-way ANOVA and subsequent S–N–K method (*n* = 5).

Based on the results of the CCK8 assay (Figure 1A) and morphology observation (Figure 1B), we tentatively chose corilagin at 100, 50, and 25 μg/ml to treat Ana-1 cells for 24 h to that ensure cell viability was ≥70%. qRT-PCR (Figure 1C) showed that there was no significant difference between corilagin-treated groups and the control group for expression of molecules involved in the TLR4 signaling pathway without ox-LDL stimulation. Thus, we used these concentrations for further experimentation.

### Stimulant Concentration and Duration of Ox-LDL Treatment on Ana-1 Cells

**Figure 2:**
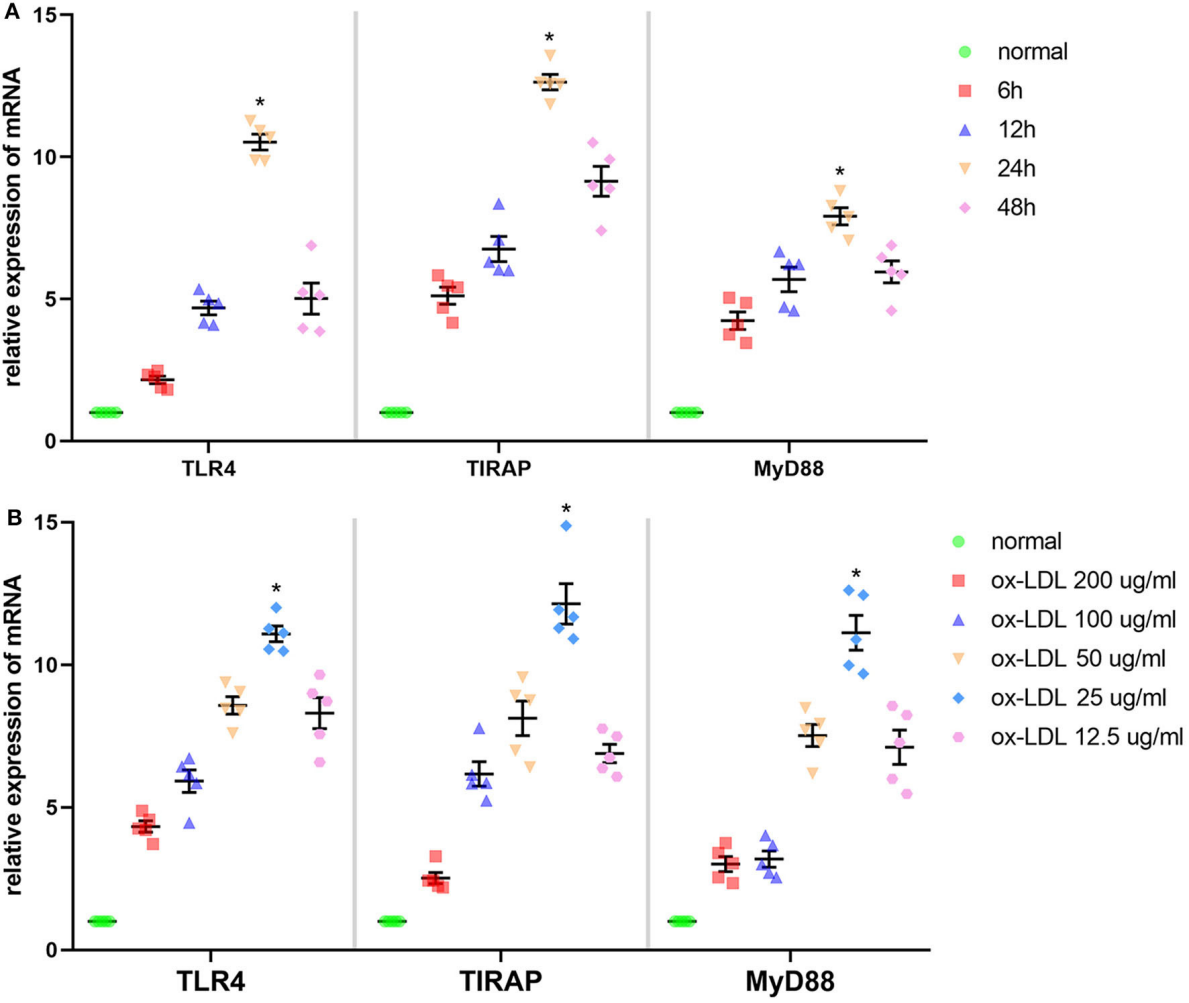
Effect of ox-LDL on mRNA expression of three molecules involved in the TLR4 signaling pathway in Ana-1 cells. **(A)** mRNA expression of TLR4, TIRAP, and MyD88 was measured by qRT-PCR. *P < 0.05 compared with time point or normal groups by one-way ANOVA and subsequent S–N–K method (*n* = 5). **(B)** mRNA expression of TLR4, TIRAP, and MyD88 was measured by qRT-PCR. *P < 0.05 compared with each concentration of ox-LDL groups or control (0 μg/ml) groups determined by one-way ANOVA and subsequent S–N–K method (*n* = 5).

According to Figure 2A, time and dose could influence the effect of ox-LDL promoting mRNA expression of the molecules involved in the TLR4 signaling pathway in Ana-1 cells. mRNA expression of TLR4, TIRAP, and MyD88 in the 24-h group was significantly higher than that at other time points or in normal groups (*P* < 0.05, *n* = 5). Also, expression at 25 μg/ml was strikingly higher than that at other concentrations or in normal groups (*P* < 0.05, *n* = 5) (Figure 2B). Finally, we stimulated Ana-1 cells with 25 μg/ml of ox-LDL for 24 h in subsequent experiments.

### Effect of Corilagin on the TLR4 Signaling Pathway After Ox-LDL Stimulation in Ana-1 Cells

**Figure 3:**
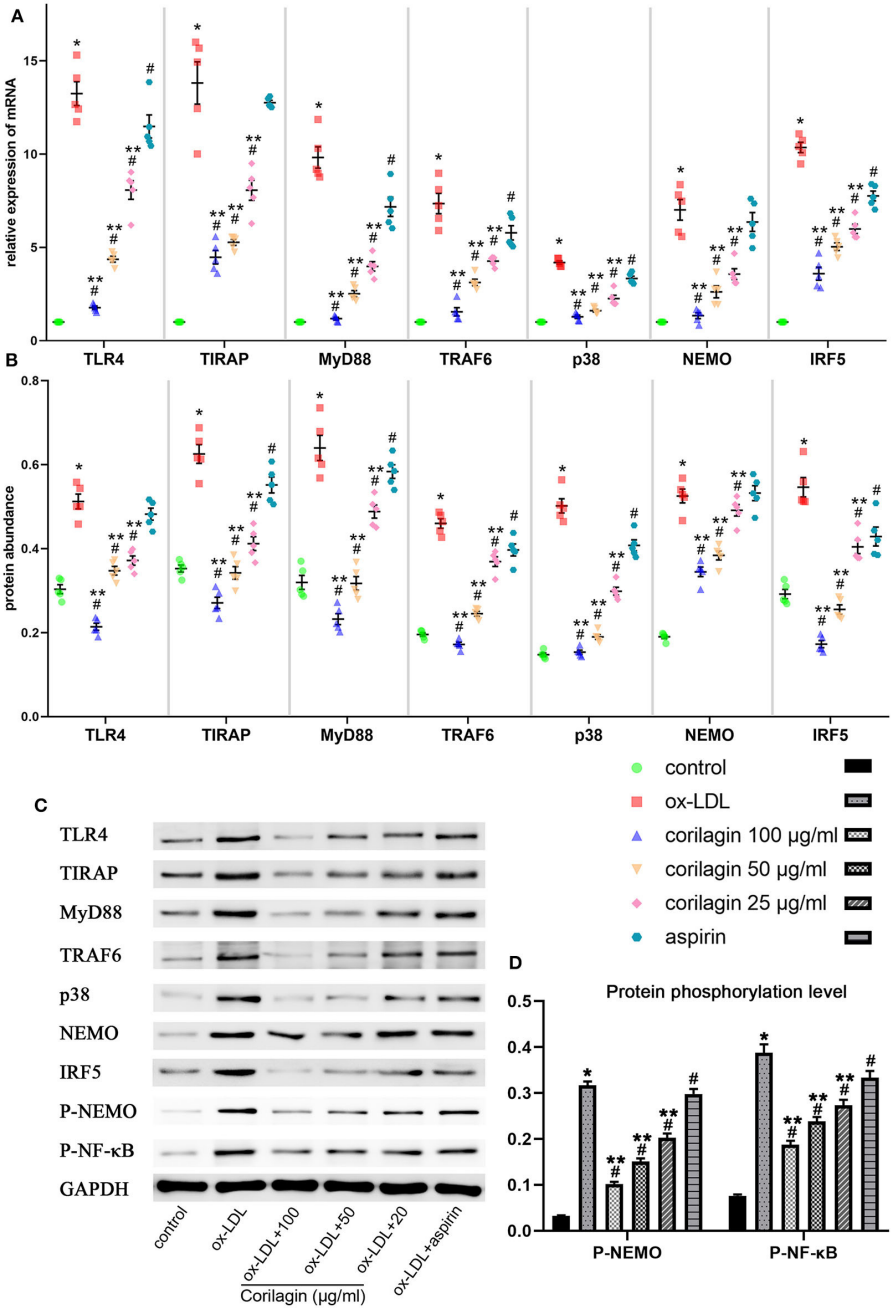
Effect of corilagin on expression of molecules in the TLR4 signaling pathway after ox-LDL stimulation in Ana-1 cells. **(A)** mRNA expression of TLR4, TIRAP, MyD88, TRAF6, p38, NEMO, and IRF5 was measured by qRT-PCR. *P < 0.05 compared with the control groups, ^#^*P* < 0.05 compared with ox-LDL groups, **P < 0.05 compared with aspirin groups determined by one-way ANOVA and subsequent S–N–K method (*n* = 5). **(B–D)** Protein expression of TLR4, TIRAP, MyD88, TRAF6, p38, NEMO, IRF5, phosphorylation of NEMO, and NK-κB was measured by Western blotting. *P < 0.05 compared with the control groups, ^#^*P* < 0.05 compared with ox-LDL groups, **P < 0.05 compared with aspirin groups determined by one-way ANOVA and subsequent S–N–K method (*n* = 5).

We measured expression of TLR4, TIRAP, MyD88, TRAF6, p38, NEMO, and IRF5 to test the anti-arteriosclerosis effect of corilagin. mRNA expression was measured by qRT-PCR (Figure 3A). An abundance of proteins was detected by Western blotting (Figures 3B,C). mRNA and protein expression of these seven molecules in 100, 50, and 25 μg/ml corilagin-treated groups was lower than that in model- and aspirin-treated groups (*P* < 0.05, *n* = 5). No significant difference was found in expression of TLR4, TIRAP, or NEMO between the aspirin group and the model group. Furthermore, we measured the phosphorylation of NEMO and NK-κB (Figures 3C,D). Similarly, the phosphorylation was enhanced by ox-LDL compared to control group and significantly inhibited by corilagin (*P* < 0.05, *n* = 5). Meanwhile, to confirm the dose-dependent manner, we treated Ana-1 cells with 100, 80, 60, 40, 20, and 10 μg/ml corilagin after ox-LDL stimulation and calculated the suppression ratio (Supplemental Figure 4). We found that the dose–response curve was “S”-shaped.

### Effect of Corilagin on the TLR4 Signaling Pathway After Ox-LDL Stimulation in TLR4-Downregulated Ana-1 Cells

**Figure 4:**
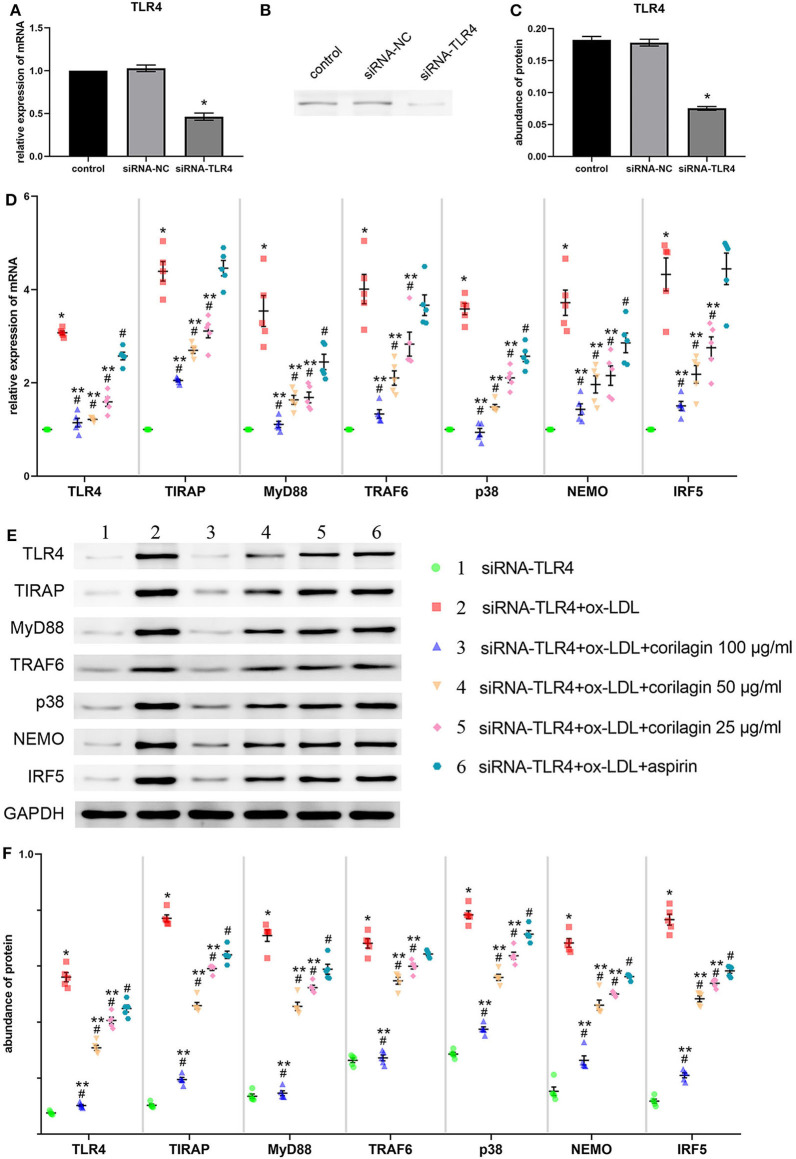
Effect of corilagin on expression of molecules involved in the TLR4 signaling pathway after ox-LDL stimulation in TLR4-downregulated Ana-1 cells. **(A)** Expression of TLR4 mRNA was measured by qRT-PCR. *P < 0.05 compared with the control group determined by the Student's *t*-test (*n* = 5). **(B,C)** Abundance of TLR4 protein was measured by Western blotting. *P < 0.05 compared with the control group determined by the Student's *t*-test (*n* = 5). **(D)** mRNA expression of molecules in the TLR4 signaling pathway was measured by qRT-PCR. *P < 0.05 compared with the siRNA-TLR4 group, ^#^*P* < 0.05 compared with model groups, **P < 0.05 compared with the aspirin group determined by one-way ANOVA and subsequent S–N–K method (*n* = 5). **(E,F)** Protein expression was measured by Western blotting. *P < 0.05 compared with the siRNA-TLR4 group, ^#^*P* < 0.05 compared with the model group (siRNA-TLR4 + ox-LDL group), **P < 0.05 compared with the aspirin group determined by one-way ANOVA and subsequent S–N–K method (*n* = 5). siRNA-TLR4: TLR4 was knocked down in Ana-1 cells by siRNA. siRNA-NC, siRNA negative control group.

We downregulated TLR4 expression in Ana-1 cells by siRNA transfection for 24 h for further research into the effect of corilagin. Compared with the control group, TLR4 expression in the downregulation (siRNA-TLR4) group fell by >50% and a significant difference was not found in the siRNA-negative control (siRNA-NC) group (Figure 4A). The abundance of TLR4 protein fell by 60% and there was no significant difference between the siRNA-NC group and the control group (Figures 4B,C).

To confirm the functional deficiency of TLR4 inhibition, we stimulated the cells with LPS and ox-LDL after TLR4 downregulation. Compared to the over 10 times overexpression effect on control, LPS or ox-LDL could only increase the mRNA expression of TLR4 three times when the TLR4 has been inhibited by siRNA (Supplemental Figure 5A). The deficiency of TLR4 also existed in protein expression (Supplemental Figure 5B).

Suppression of the TLR4 signaling pathway by corilagin (100, 50, and 25 μg/ml) was significant and more remarkable than that elicited by aspirin (*P* < 0.05, *n* = 5) (Figure 4D). No significant difference was found in expression of TIRAP, TRAF6, or IRF5 between the aspirin group and the model group. Protein expression was measured by Western blotting (Figures 4E,F). All three concentrations of corilagin could continue to inhibit the high expression of the TLR4 signaling pathway caused by ox-LDL, and their effect was much more prominent than that of aspirin (*P* < 0.05, *n* = 5).

### Effect of Corilagin on the TLR4 Signaling Pathway After Ox-LDL Stimulation in TLR4-Upregulated Ana-1 Cells

**Figure 5:**
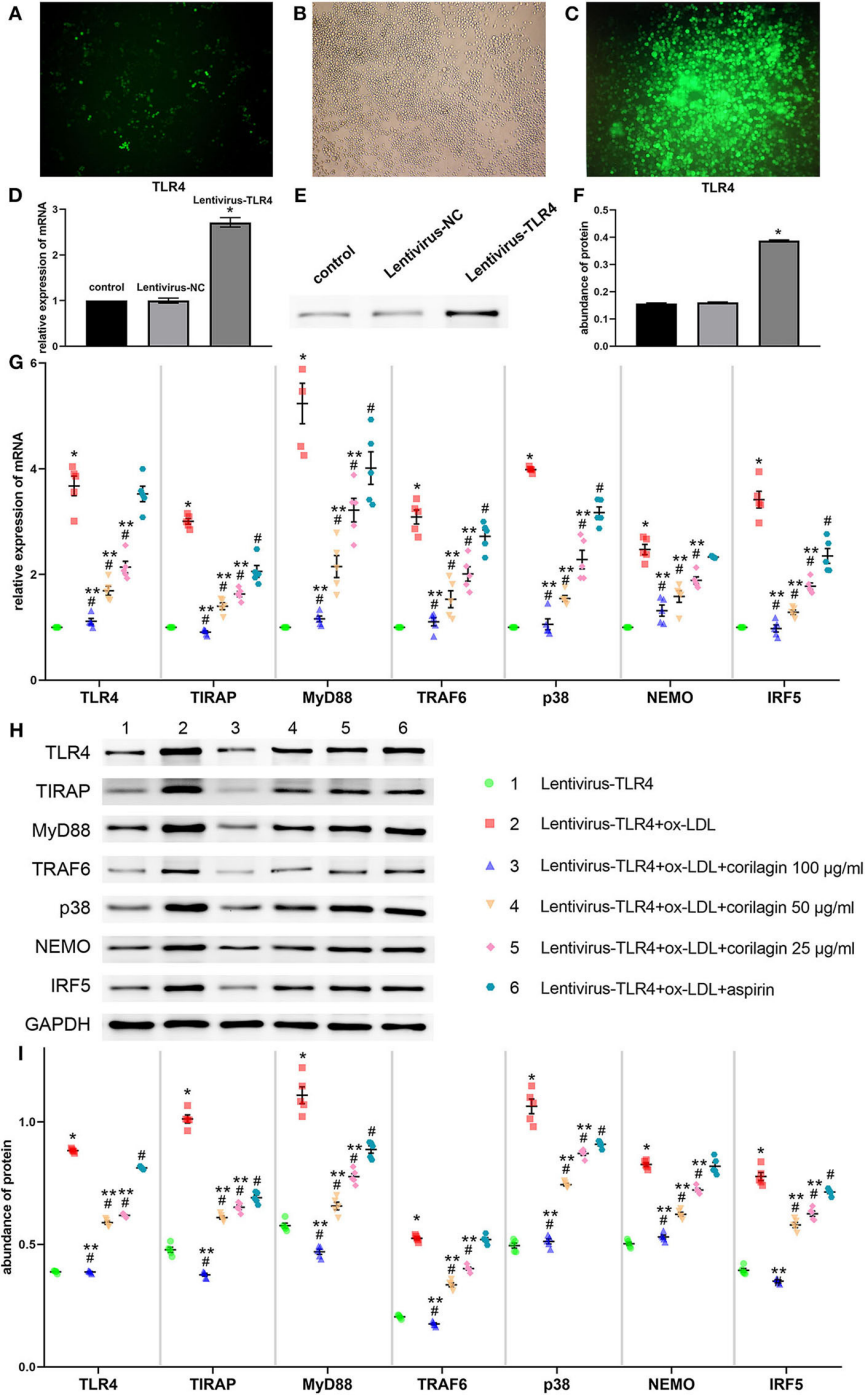
Effect of corilagin on expression of molecules involved in the TLR4 signaling pathway after ox-LDL stimulation in TLR4-upregulated Ana-1 cells. **(A,B)** Upregulation of TLR4 expression in Ana-1 cells via the lentiviral vector. GFP expression was observed with a fluorescence microscope after the lentivirus had been introduced into Ana-1 cells for 72 h. **(C)** After elimination by puromycin for 7 days, fluorescence was observed. **(D)** Expression of TLR4 mRNA was measured by qRT-PCR. *P < 0.05 compared with the control group determined by the Student's *t*-test (*n* = 5). **(E,F)** Expression of TLR4 protein was measured by Western blotting. *P < 0.05 compared with the control group determined by the Student's *t*-test (*n* = 5). **(G)** mRNA expression of molecules involved in the TLR4 signaling pathway was measured by qRT-PCR. *P < 0.05 compared with the lentivirus-TLR4 group, ^#^*P* < 0.05 compared with the model group (lentivirus-TLR4 + ox-LDL group), **P < 0.05 compared with the aspirin group determined by one-way ANOVA and subsequent S–N–K method (*n* = 5). **(H,I)** Abundance of the protein of TLR4, TIRAP, MyD88, TRAF6, p38, NEMO, and IRF5 was measured by Western blotting. *P < 0.05 compared with the lentivirus-TLR4 group, ^#^*P* < 0.05 compared with the model group, **P < 0.05 determined by one-way ANOVA and subsequent S–N–K method (*n* = 5).

We constructed the TLR4 lentiviral vector and transduced Ana-1 cells *in vitro* to upregulate TLR4 expression. Seventy-two hours after infection, we observed fluorescence (GFP) in Ana-1 cells under the fluorescence microscope (Figures 5A,B). Sifting with puromycin for 7 days, almost 100% of Ana-1 cells expressed GFP (Figure 5C). The effect of the lentiviral vector and corilagin on the TLR4 signaling pathway was determined by qRT-PCR and Western blotting. mRNA expression of TLR4 in the upregulation group was 180% higher than that in the control group, and no significant difference was found in the lentivirus-negative control (lentivirus-NC) group (*P* < 0.05, *n* = 5). The abundance of TLR4 protein was increased to 150%, and there was no significant difference between the lentivirus-NC group and control group (*P* < 0.05, *n* = 5) (Figures 5D–F). Suppression of the TLR4 signaling pathway by corilagin (100, 50, and 25 μg/ml) was significant and more remarkable than that elicited by aspirin (*P* < 0.05, *n* = 5). No significant difference was found in expression of TLR4 or NEMO between the aspirin group and the model group (Figure 5G). Protein expression was measured by Western blotting (Figures 5H,I). All three concentrations of corilagin could continue to inhibit high expression of the molecules involved in the TLR4 signaling pathway caused by ox-LDL, and showed a much more prominent effect than that elicited by aspirin (*P* < 0.05, *n* = 5). No significant difference was found in expression of TRAF6 or NEMO between the aspirin group and the model group.

### Effect of Corilagin on the TLR4 Signaling Pathway After Ox-LDL Stimulation in MPMs

**Figure 6:**
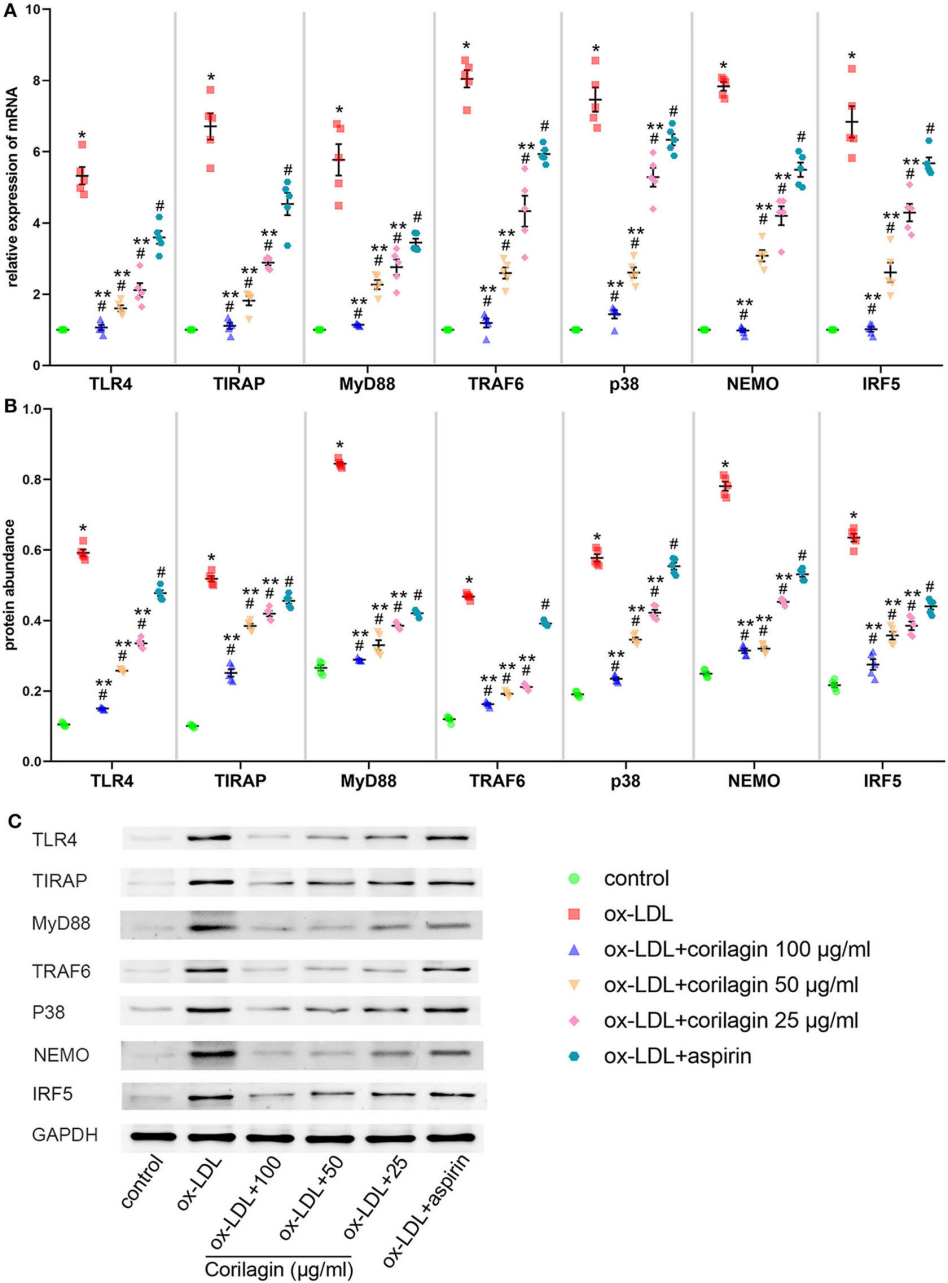
Effect of corilagin on the TLR4 signaling pathway after ox-LDL stimulation in MPMs. **(A)** mRNA expression of TLR4, TIRAP, MyD88, TRAF6, p38, NEMO, and IRF5 was measured by qRT-PCR. *P < 0.05 compared with the control group, ^#^*P* < 0.05 compared with the ox-LDL group, **P < 0.05 compared with the aspirin group determined by one-way ANOVA and subsequent S–N–K method (*n* = 5). **(B,C)** Protein expression was measured by Western blotting. *P < 0.05 compared with the control group, ^#^*P* < 0.05 compared with the ox-LDL group, **P < 0.05 compared with the aspirin group determined by one-way ANOVA and subsequent S–N–K method (*n* = 5).

MPMs were extracted from C57BL/6 mice. Upon stimulation with ox-LDL, TLR4 signaling in MPMs showed notably high expression (*P* < 0.05, *n* = 5) (Figure 6). Then, we treated MPMs with corilagin or aspirin, and observed the inhibitory effect by qRT-PCR and Western blotting. Significant inhibition of mRNA (Figure 6A) and protein (Figures 6B,C) expression in the TLR4 signaling pathway was found in 100, 50, and 25 μg/ml corilagin-treated groups compared with that in the control group or aspirin group (*P* < 0.05, *n* = 5).

### Effect of Corilagin on TLR2 in Ana-1 Cells

As TLR2 and TLR4 shared many signaling pathways including molecules such as TIRAP and MyD88 (33), it is necessary to eliminate the confusion caused by TLR2. We treated Ana-1 cells with corilagin after ox-LDL stimulation and measured the expression of TLR2 and TLR4. Compared to TLR4, the increased expression of TLR2 was inconspicuous. Corilagin could inhibit the overexpression of TLR4 much more effectively than TLR2 (Supplemental Figure 6).

### Effect of Corilagin on the TLR4 Signaling Pathway in the Rat Model of PAD

**Figure 7:**
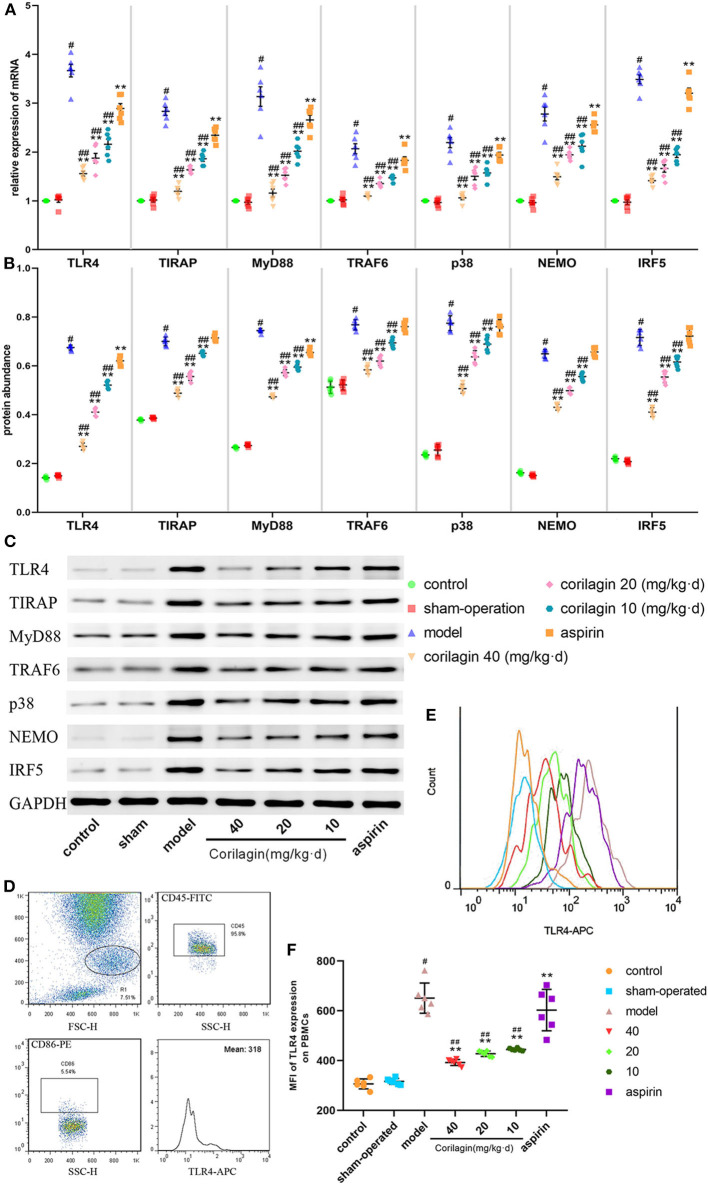
Effect of corilagin on the TLR4 signaling pathway in PBMCs from a rat model of PAD. **(A)** mRNA expression of TLR4, TIRAP, MyD88, TRAF6, p38, NEMO, and IRF5 was measured by qRT-PCR. ^#^*P* < 0.05 compared with the control group, **P < 0.05 compared with the model group, ^*##*^*P* < 0.05 compared with the aspirin group determined by one-way ANOVA and subsequent S–N–K method (*n* = 6). **(B,C)** Protein expression was measured by Western blotting. ^#^*P* < 0.05 compared with control groups, **P < 0.05 compared with the model group, ^*##*^*P* < 0.05 compared with aspirin groups determined by one-way ANOVA and subsequent S–N–K method (*n* = 6). **(D–F)** TLR4 expression on PBMC membranes from a rat model of PAD tested by FCM. ^#^*P* < 0.05 compared with the control group, **P < 0.05 compared with the model group, ^*##*^*P* < 0.05 compared with the aspirin group determined by one-way ANOVA and subsequent S-N-K method (*n* = 6).

Expression of molecules in the TLR4 signaling pathway in PBMCs was measured by qRT-PCR and Western blotting (Figures 7A–C). Expression in the model group was significantly higher than that in control and sham-operated groups (*P* < 0.05, *n* = 6). Corilagin continued to show effective inhibition of the TLR4 signaling pathway at mRNA and protein levels, and it was much higher than that elicited by aspirin (*P* < 0.05, *n* = 6). Only in terms of the protein abundance of TLR4 and MyD88 was a significant difference found between the aspirin group and control group. We measured TLR4 expression on cell membranes by FCM to verify our results: we observed the same trend (Figures 7D–F).

**Figure 8:**
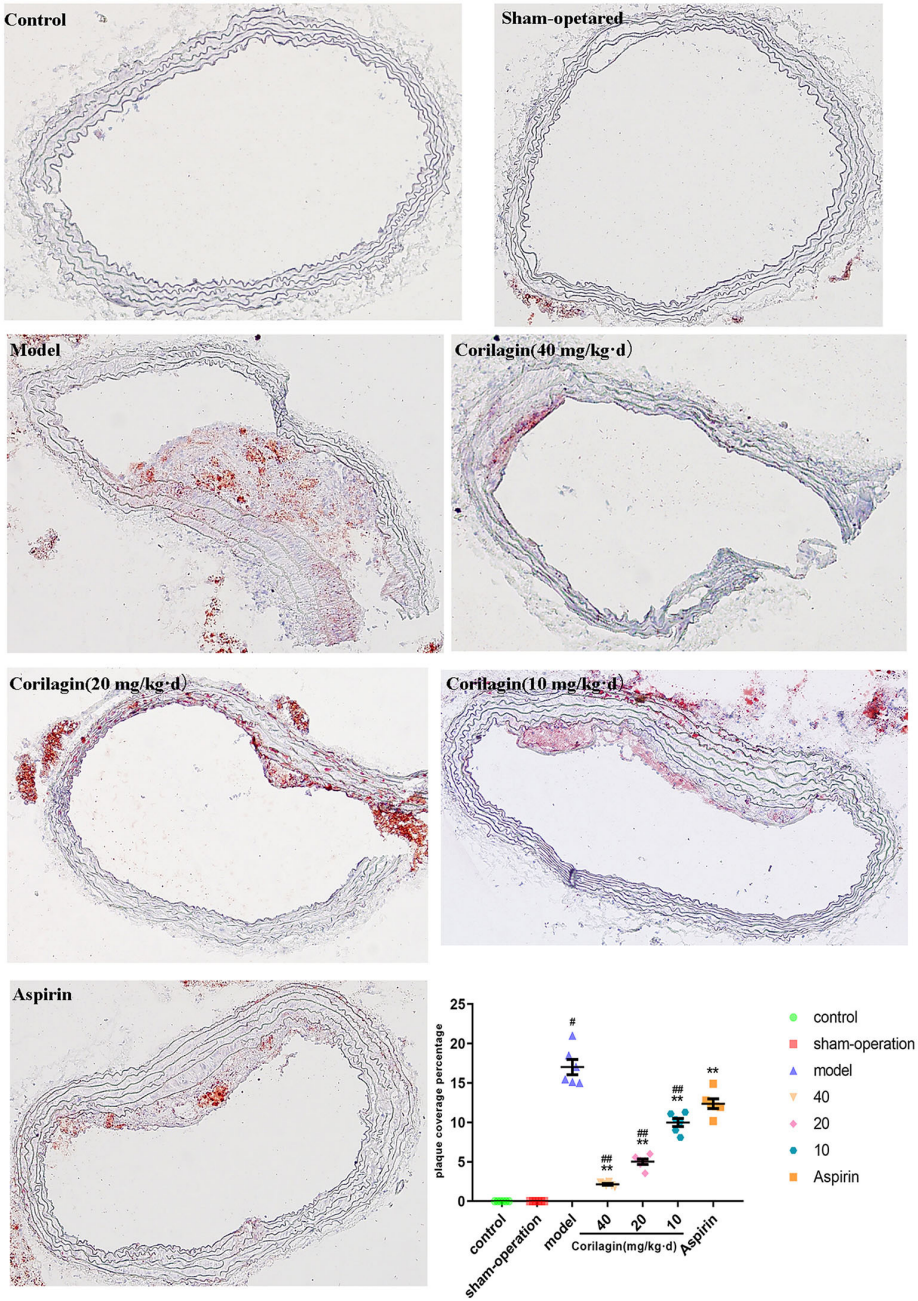
Effect of Corilagin on percentage plaque coverage in femoral arteries from PAD rat model. ^#^*P* < 0.05 compared with control group, **P < 0.05 compared with model group, ^*##*^*P* < 0.05 compared with aspirin group determined by one-way ANOVA method and subsequent S–N–K method (*n* = 6).

Atherosclerosis in the femoral artery in a rat model of PAD was determined by observing pathology sections after Oil Red O staining (Figure 8). We observed sclerotic plaques in all slices except in control and sham-operated groups. However, the percentage plaque coverage in corilagin groups was significantly lower than that in the model group and aspirin group (*P* < 0.05, *n* = 6).

**Figure 9:**
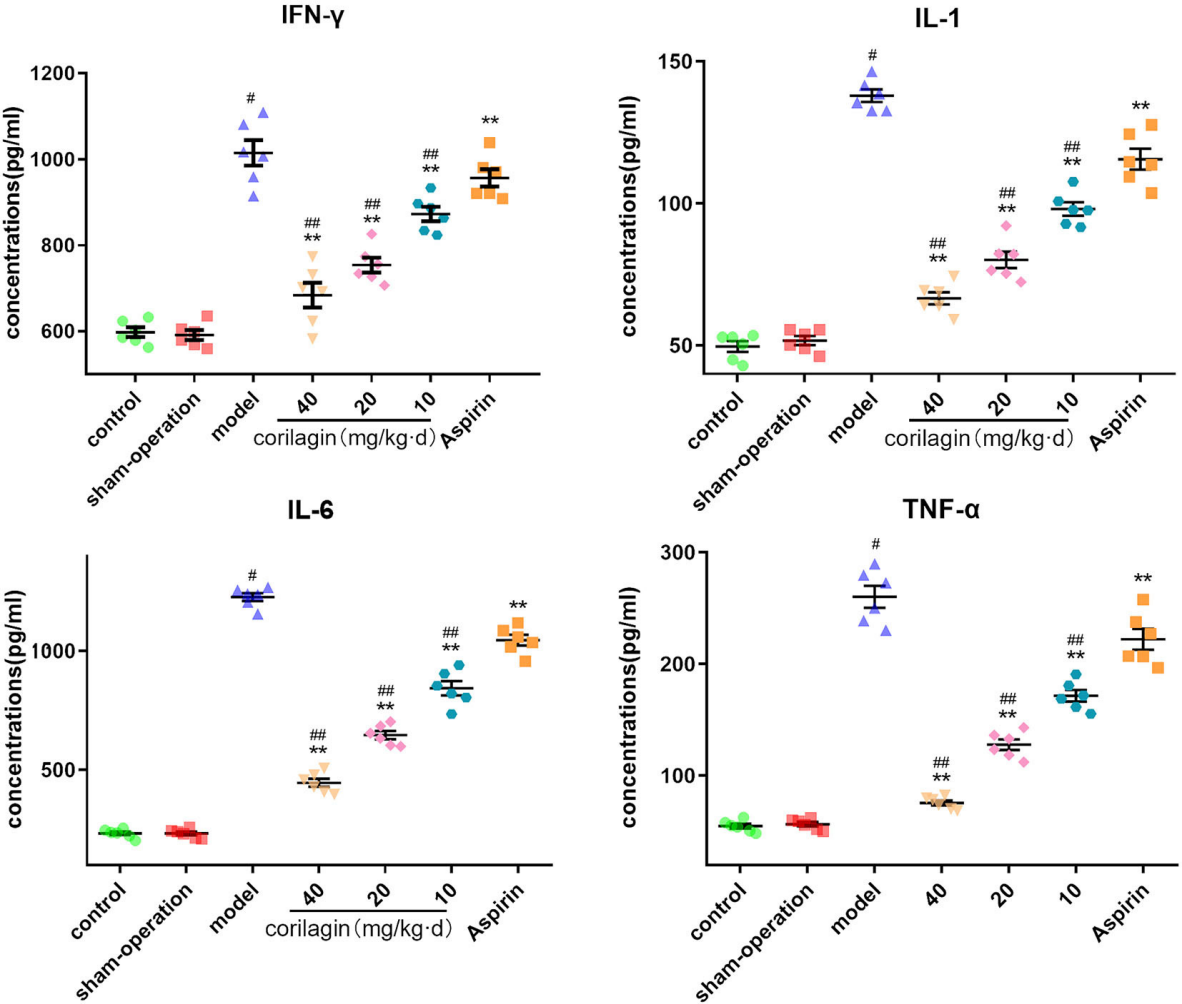
Effect of corilagin on inflammatory factor in plasma from a rat model of PAD tested by ELISA. ^#^*P* < 0.05 compared with the control group, **P < 0.05 compared with the model group, ^*##*^*P* < 0.05 compared with the aspirin group determined by one-way ANOVA and subsequent S–N–K method (*n* = 6).

Moreover, we measured expression of some major cytokines in plasma in the rat model of PAD by ELISA (Figure 9). Although the level of IFN-γ, IL-1β, IL-6, and TNF-α in corilagin groups was higher than that in control and sham-operated groups, it was significantly lower than that in the model group and aspirin group (*P* < 0.05, *n* = 6).

## Discussion

In recent decades, increasing evidence has suggested that atherosclerosis is a chronic inflammatory disease involving the adaptive immune and innate immune systems (34). Initially, LDL infiltrates were deposited under the arterial endothelium and modified partially by myeloperoxidase, lipoxygenase, or reactive oxygen species to become ox-LDL (35). As one of the most important promotors of the innate inflammatory response, ox-LDL can act on cells in the lesion area, such as macrophages, which differentiate from monocytes stimulated by macrophage-colony stimulating factor and granulocyte–macrophage colony stimulating factor in the intima. Circulating monocytes are recruited to these sites, and differentiate and proliferate constantly, which ensures that the atherosclerotic area is filled with monocyte-derived macrophages (36).

Macrophages have a complicated role in the initiation and progression of atherosclerotic plaques in various ways. Continuous uptake of lipoproteins via scavengers or CD36 (37) leads them to transform into foam cells and, finally, die in the core area and release cholesterol crystals. We do not classify these macrophages as “M1” or “M2” cells because many of them do not meet the criteria, but instead they have a novel phenotype for characteristics that are induced by oxidized phospholipids (38).

Ox-LDL as an endogenous ligand for TLR4. It can activate macrophages and then activate the innate immune system. LDL oxidized by lipoprotein-associated phospholipase A2 (Lp-PLA2), lysophosphatidylcholine, and oxidized non-esterified fatty acids are generated and can also activate the innate immune system (39). Other ligands for TLR4 are present in atherosclerotic plaques, such as endogenous heat-shock proteins, exogenous lipopolysaccharide, or other bacterial toxins and glycoproteins from viral envelopes (40–42). Unfortunately, attempts to counteract coronary heart disease by preventing activation of the innate immune system by antioxidants (43) or selective inhibitors of Lp-PLA2 (44, 45) have failed. Therefore, we tried to find a different target and strategy.

TLR4 is overexpressed by mononuclear macrophages in mice as well as in the circulation and lipid-rich atherosclerotic lesions of humans (19, 46). Some scholars have suggested that an absence of TLR4 or MyD88 could lead to reduction of formation of atherosclerotic plaques in mice (47). We found similar results: *In vitro*, stimulating Ana-1 cells or MPMs with ox-LDL resulted in overexpression of TLR4 and the downstream molecules TIRAP, MyD88, TRAF6, p38, NEMO, and IRF5. *In vivo*, expression of TLR4, TIRAP, MyD88, TRAF6, p38, NEMO, and IRF5 in PBMCs in the PAD model group was higher than that in the control group and sham-operation group. These results demonstrated the high expression of TLR4 by mononuclear macrophages in the circulation of rats with atherosclerosis, and suggested that there might be a positive-feedback mechanism among TLR4 and its downstream molecules. Some scholars have shown that downstream molecules such as MyD88 and TRAF6 also have key effects on atherosclerosis. Mice given ApoE^−/−^Myd88^−/−^CD4^+^ T cells have been shown to exhibit reduced T-helper (Th)17 immunity and plaque growth (48). Inhibition of CD40-TRAF6 expression may abolish atherosclerosis and confer plaque fibrosis in ApoE^−/−^ mice by inducing differentiation of macrophages toward a M2 signature (49). Thus, we attempted to find a new therapeutic drug for atherosclerosis by reducing TLR4 signaling.

Our research team has shown that corilagin exerts an anti-inflammatory effect in sepsis through downregulation of expression of TLR4, MyD88, TRIF, and TRAF6 in the TLR4 signaling pathway (31). The present study is the first to investigate if corilagin could cure atherosclerosis and its possible mechanism of action.

We incubated Ana-1 cells or MPMs with corilagin for 24 h, which had been stimulated by ox-LDL in advance. Corilagin elicited a prominent reduction of the mRNA and protein expression of TLR4, TIRAP, MyD88, TRAF6, p38, NEMO, and IRF5. Aspirin (the most commonly used anti-inflammatory agent) can inhibit TLR4 expression in C26 colon cancer cells (26). However, its efficacy was much weaker than that of corilagin in the present study. Compared with that at a low concentration, corilagin at a higher concentration (in the range of maximum non-toxic concentration) might have better pharmacologic properties.

To further verify the effect of corilagin, we downregulated TLR4 expression in Ana-1 cells to alter the sensitivity to ox-LDL. The latter could continue to promote the transcription and translation of TLR4, but corilagin showed significant inhibition of the TLR4 signaling pathway. When we upregulated TLR4 expression in Ana-1 cells, corilagin could restrain the TLR4 signaling pathway at the initial point of upregulation of TLR4 expression. Hence, it is rational to suggest that the anti-inflammatory and anti-atherosclerotic effect of corilagin was associated with ox-LDL stimulation. On account of the major role ox-LDL in atherosclerosis, corilagin exhibited tremendous potential. However, how corilagin affects the TLR4 signaling pathway, whether another mechanism is involved, and whether TLR4 (as the central upstream molecule in the TLR4 signaling pathway) might also regulate the downstream molecules TIRAP, MyD88, TRAF6, p38, NEMO, and IRF5 are worth pondering.

It has been shown that ox-LDL-containing plasma induced IL-1β production in a CD36-, TLR2-, TLR4-, and TLR6-dependent manner in primary human monocytes (23). In addition, it is well studied that TLR2 and TLR4 shared many signaling pathways (33). Therefore, we compared the effect of corilagin on TLR2 and TLR4 to determine its target. Results showed that corilagin was much more effective on TLR4 than TLR2. This is another clue to the conjecture that corilagin might aim at TLR4 but not TLR2 and thus inhibit the signal pathway.

We wished to confirm the anti-atherosclerotic effect of corilagin *in vivo*. We destroyed endothelial cells in the femoral artery of rats. The extent of atherosclerotic lesions in the femoral artery in rats given corilagin by gavage was remarkably smaller than that for rats in the model group and aspirin group. This was strong evidence of the inhibitory effect upon plaque development by corilagin. PBMC extraction followed by qRT-PCR and Western blotting provided more evidence of suppression of the TLR4 signaling pathway by corilagin. FCM used to measure TLR4 expression on cell-surface membranes also showed an obvious reduction in expression by corilagin. Variations in the lesion area among experimental groups and the control group were in accordance with expression of molecules involved in the TLR4 signaling pathway. Those data provided indirect (but clear and compelling) evidence that corilagin could restrain the development of atherosclerotic plaques by inhibiting the TLR4 signaling pathway *in vivo*.

**Figure 10:**
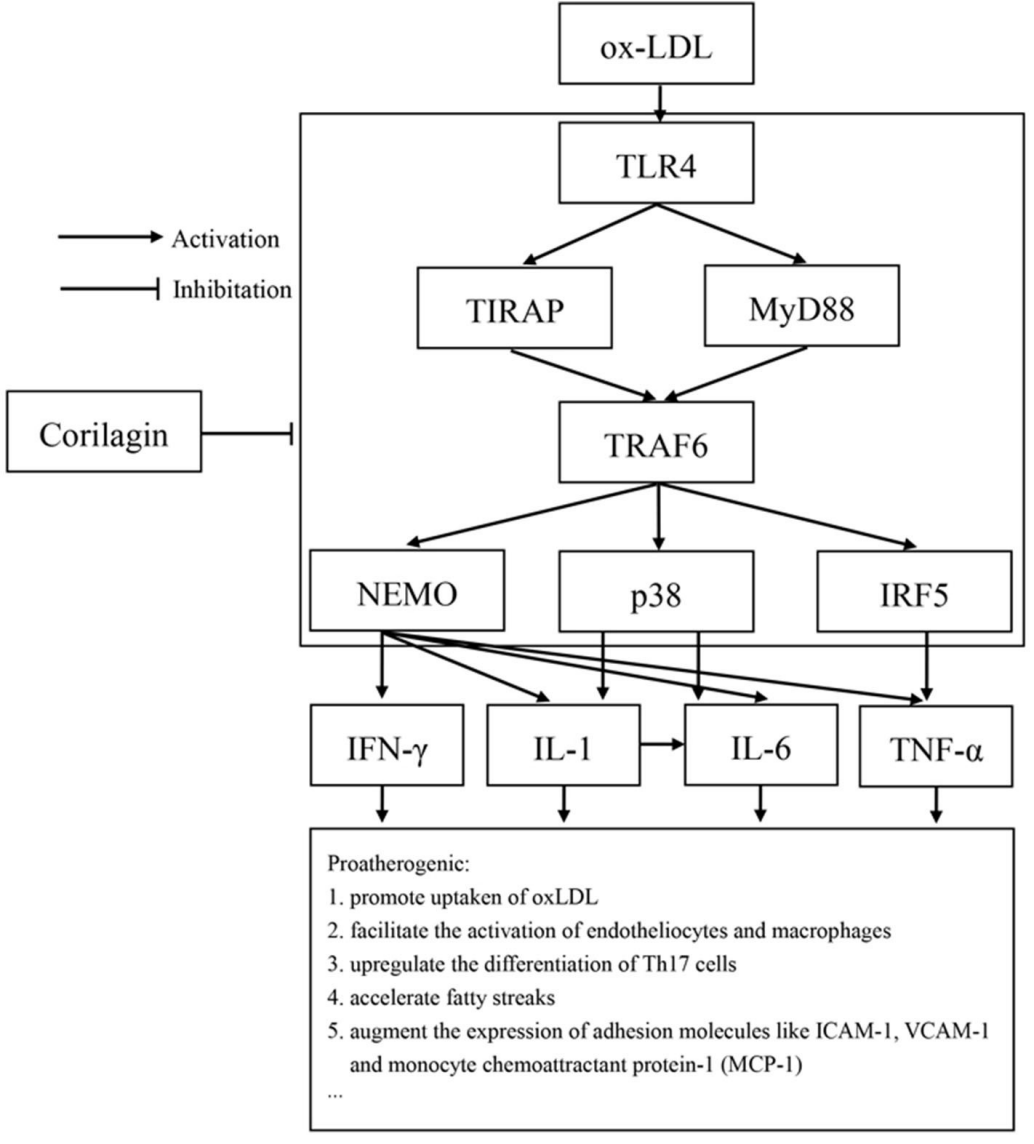
TLR4 had an important proatherogenic role in monocytes/macrophages. Corilagin effectively inhibited the TLR4 signaling pathway to alleviate atherosclerosis. ICAM-1: intercellular adhesion molecule 1; VCAM-1: vascular adhesion molecule 1.

Studies have demonstrated the importance of several inflammatory factors in the entire course of atherosclerosis. IFN-γ and TNF-α have been found to affect CD4^+^ Th1 cells and then cause the acceleration of inflammation, lesion growth, and plaque instability (50, 51). Release of matrix metalloproteinases by endothelial cells, smooth muscle cells, and monocytes/macrophages is closely related to IL-1β expression. IL-6 is considered to be a causal risk for atherothrombotic disease, and its expression can also be regulated by IL-1β (52, 53). In our study, expression of IFN-γ, IL-1β, IL-6, and TNF-α increased in the model group and was downregulated by corilagin. Considering that expression of the genes of these cytokines can be regulated by NK-κB, p38, or IRF-5, we speculate that the reduction was due to inhibition of the TLR4 signaling pathway by corilagin (Figure 10). To prove this hypothesis, the experiments with TLR4 knockout rats *in vivo* must be done.

Our study had three main strengths. We explore, for the first time, the anti-atherosclerosis effect of corilagin *in vitro* and *in vivo*. Second, we found that the TLR4 signaling pathway may be a target of corilagin, that downstream molecules would also be affected, and, finally, that expression of some important molecules (IFN-γ, IL-1β, IL-6, and TNF-α) was inhibited. Third, we found that extracellular hypo-osmosis caused by sterile water could destroy endothelial cells effectively, and that this method was easier to operate than guidewire puncture.

Our study had two main limitations. The exact mechanism by which corilagin affects the TLR4 signaling pathway was not studied. Second, whether corilagin could inhibit downstream molecules in other ways was not studied.

## Conclusions

We clarified the anti-atherosclerosis efficacy of corilagin on monocytes/macrophages by inhibiting the TLR4 signaling pathway and then suppressing the inflammatory response *in vitro* and *in vivo*. However, more direct evidence is needed, and we will continue to focus on this aspect. We consider that corilagin has enormous potential to control PAD in humans.

## Data Availability Statement

All datasets presented in this study are included in the article/Supplementary Material.

## Ethics Statement

The animal study was reviewed and approved by Tongji Medical College, HUST Institutional Animal Care and Use Committee.

## Author Contributions

LZ: project administration, study conceptualization, and methodology. YD: study conceptualization, supervision, and software procurement. YLi: data curation and writing—review and editing. YuW: methodology, data curation, and writing—preparation of original draft. YC: data curation/analyses. YaW: project administration. SZ, PL, and ZC: investigation. PS: software procurement. LL: data curation. YLu: data analyses. All authors contributed to the article and approved the submitted version.

## Conflict of Interest

The authors declare that the research was conducted in the absence of any commercial or financial relationships that could be construed as a potential conflict of interest.

## Supplementary Material

Supplemental Figure 1 Downregulation efficiency of siRNA. The efficiency was checked by qRT-PCR and Western blotting. **(A)** mRNA expression of TLR4 was measured by qRT-PCR. **P* < 0.05 compared with any other groups determined by one-way ANOVA method and subsequent S-N-K method (*n* = 5). **(B,C)** Protein expression of TLR4 was measured by Western blotting. **P* < 0.05 compared with any other groups determined by one-way ANOVA method and subsequent S–N–K method (*n* = 5).

Supplemental Figure 2 Effect of corilagin, aspirin and TAK-242 on Ana-1 after ox-LDL stimulation. **(A)** mRNA expression of TLR4 and MyD88 was measured by qRT-PCR. **P* < 0.05 compared with model (ox-LDL), aspirin and TAK-242 groups; ^#^*P* < 0.05 compared with the model group. No significant difference were found between TAK-242 and model groups determined by one-way ANOVA method and subsequent S–N–K method (*n* = 5). **(B,C)** protein expression of TLR4 and MyD88 was measured by Western blotting. **P* < 0.05 compared with model, aspirin and TAK-242 groups; ^#^*P* < 0.05 compared with the model group. No significant difference were found between TAK-242 and model groups determined by one-way ANOVA method and subsequent S–N–K method (*n* = 5).

Supplemental Figure 3 Concentrations and mortality curve. We treated SD rats with corilagin from 5 to 160 mg/kg·d. No deaths occurred in 5, 10, 20, and 40 mg/kg·d groups (*n* = 6). One deaths occurred in 80 mg/kg·d group (*n* = 6), 4 deaths in 160 mg/kg·d group (*n* = 6), five deaths in 320 mg/kg·d group (*n* = 6).

Supplemental Figure 4 Dose-dependent curve. Ana-1 cells were treated with 100, 80, 60, 40, 20, and 10 μg/ml corilagin after ox-LDL stimulation. mRNA expression of TLR4 was measured by qRT-PCR. Suppression ratio were calculated according to the results of qRT-PCR. The curve was “S”-shaped.

Supplemental Figure 5 Functional deficiency of TLR4 inhibition caused by siRNA. We stimulated the cells with LPS and ox-LDL after TLR4 downregulation. **(A)** mRNA expression of TLR4 was measured by qRT-PCR. **P* < 0.05 compared with control group; ^#^*P* < 0.05 compared with siRNA group; ***P* < 0.05 compared with LPS group; ^Δ^*P* < 0.05 compared with ox-LDL group determined by one-way ANOVA method and subsequent S–N–K method (*n* = 5). **(B)** Protein expression was measured by Western blotting. **P* < 0.05 compared with control group; ^#^*P* < 0.05 compared with siRNA group; **P* < 0.05 compared with LPS group; ^Δ^*P* < 0.05 compared with ox-LDL group determined by one-way ANOVA method and subsequent S–N–K method (*n* = 5).

Supplemental Figure 6 Changes of TLR4 and TLR2 in atherosclerosis cell models. We treated Ana-1 cells with corilagin after ox-LDL stimulation. **(A)** mRNA expression was measured by qRT-PCR. **P* < 0.05 compared with control group; ^#^*P* < 0.05 compared with the model (ox-LDL) group determined by one-way ANOVA method and subsequent S–N–K method (*n* = 5). **(B,C)** Protein expression was measured by Western blotting. **P* < 0.05 compared with control group; ^#^*P* < 0.05 compared with the model (ox-LDL) group determined by one-way ANOVA method and subsequent S–N–K method (*n* = 5).
