# Supplementary figures and images for: Corilagin Ameliorates Atherosclerosis in Peripheral Artery Disease via the Toll-Like Receptor-4 Signaling Pathway in vitro and in vivo
Source: Front Immunol. 2020 Aug 6;11:1611. doi: 10.3389/fimmu.2020.01611 (PMC7424006; doi:10.3389/fimmu.2020.01611)

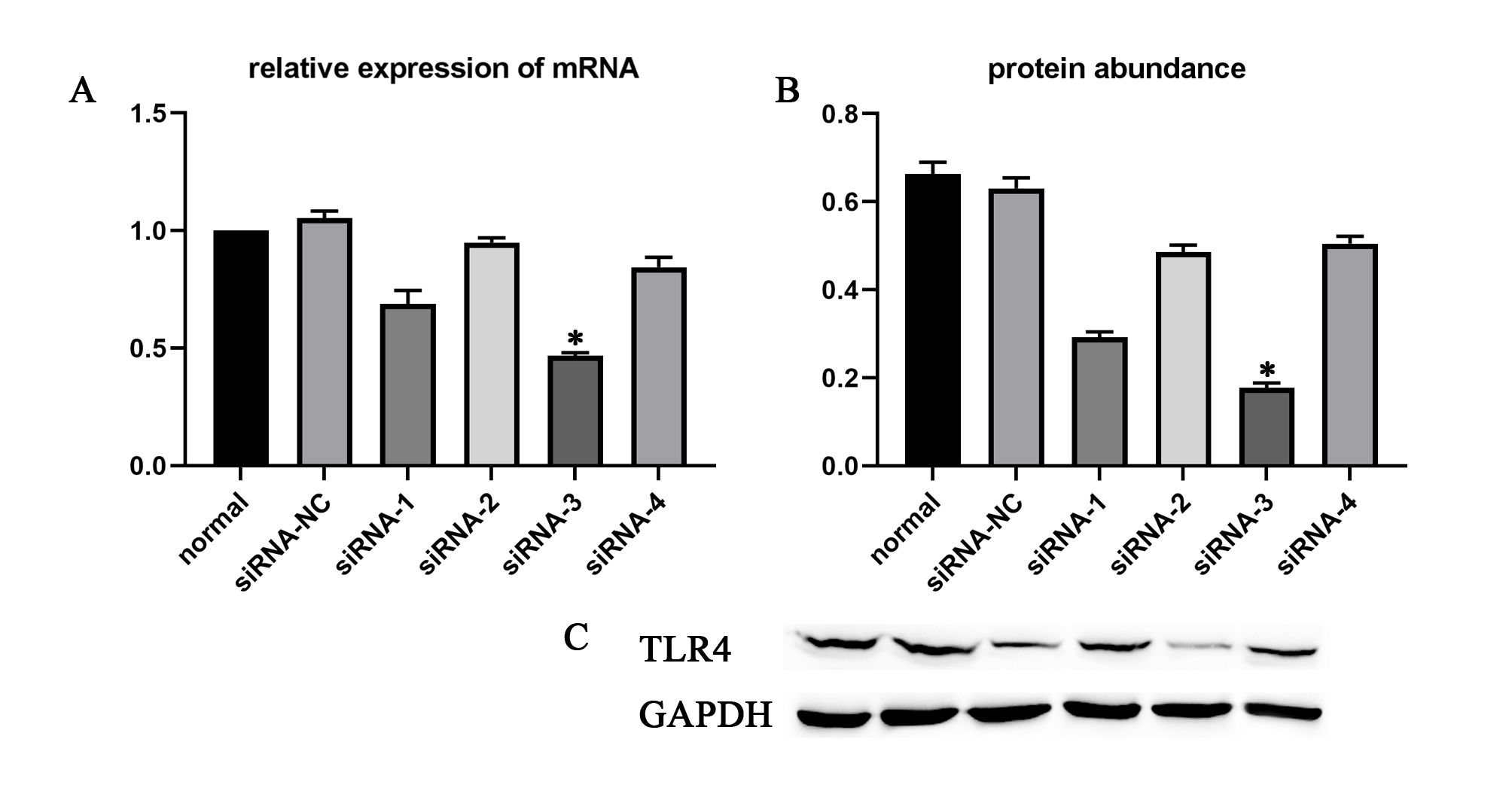

Supplement: Supplemental Figure 1 — Downregulation efficiency of siRNA. The efficiency was checked by qRT-PCR and Western blotting. (A) mRNA expression of TLR4 was measured by qRT-PCR. *P < 0.05 compared with any other groups determined by one-way ANOVA method and subsequent S-N-K method (n = 5). (B,C) Protein expression of TLR4 was measured by Western blotting. *P < 0.05 compared with any other groups determined by one-way ANOVA method and subsequent S–N–K method (n = 5). [file Image_1.JPEG]

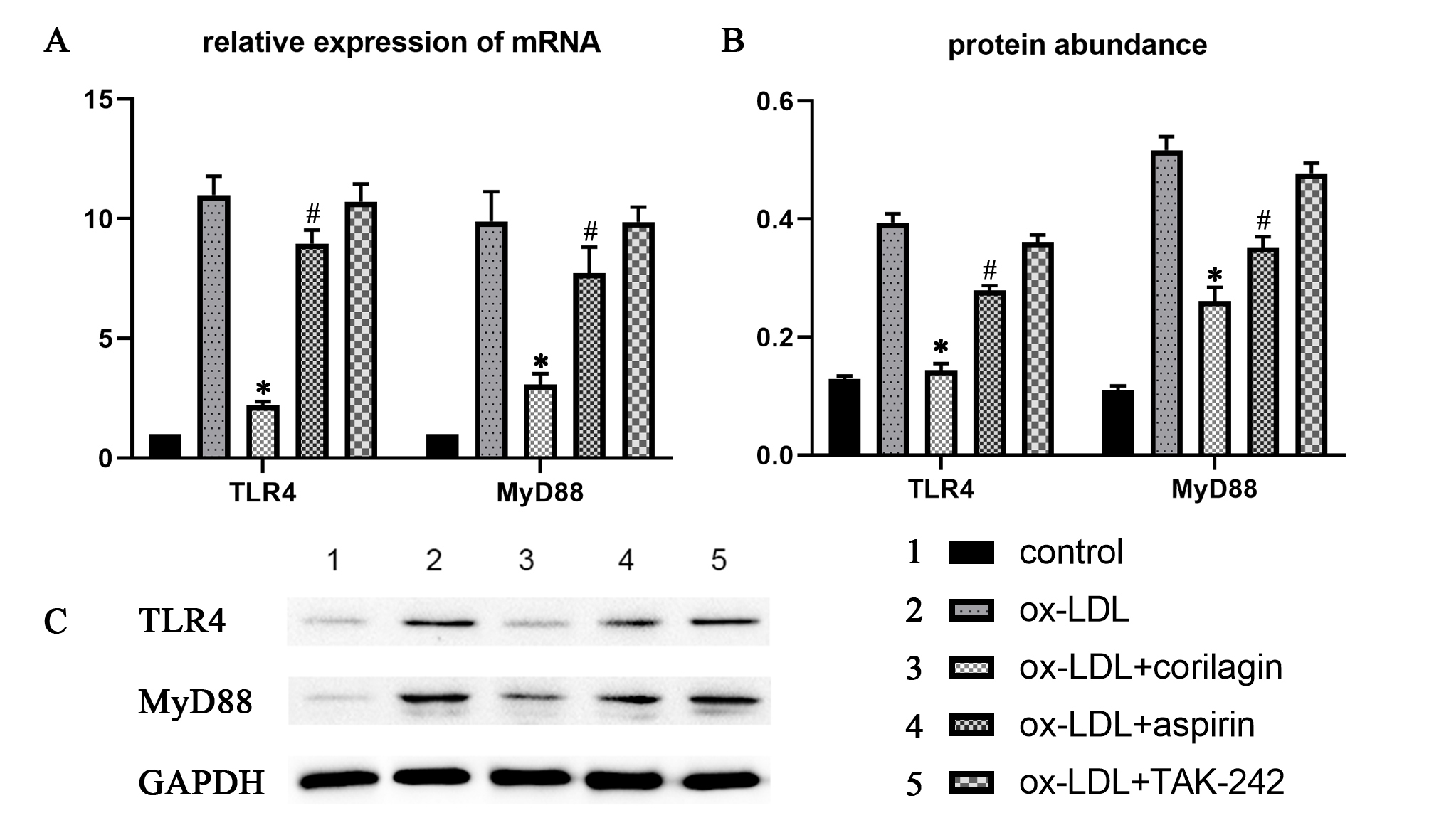

Supplement: Supplemental Figure 2 — Effect of corilagin, aspirin and TAK-242 on Ana-1 after ox-LDL stimulation. (A) mRNA expression of TLR4 and MyD88 was measured by qRT-PCR. *P < 0.05 compared with model (ox-LDL), aspirin and TAK-242 groups; #P < 0.05 compared with the model group. No significant difference were found between TAK-242 and model groups determined by one-way ANOVA method and subsequent S–N–K method (n = 5). (B,C) protein expression of TLR4 and MyD88 was measured by Western blotting. *P < 0.05 compared with model, aspirin and TAK-242 groups; #P < 0.05 compared with the model group. No significant difference were found between TAK-242 and model groups determined by one-way ANOVA method and subsequent S–N–K method (n = 5). [file Image_2.JPEG]

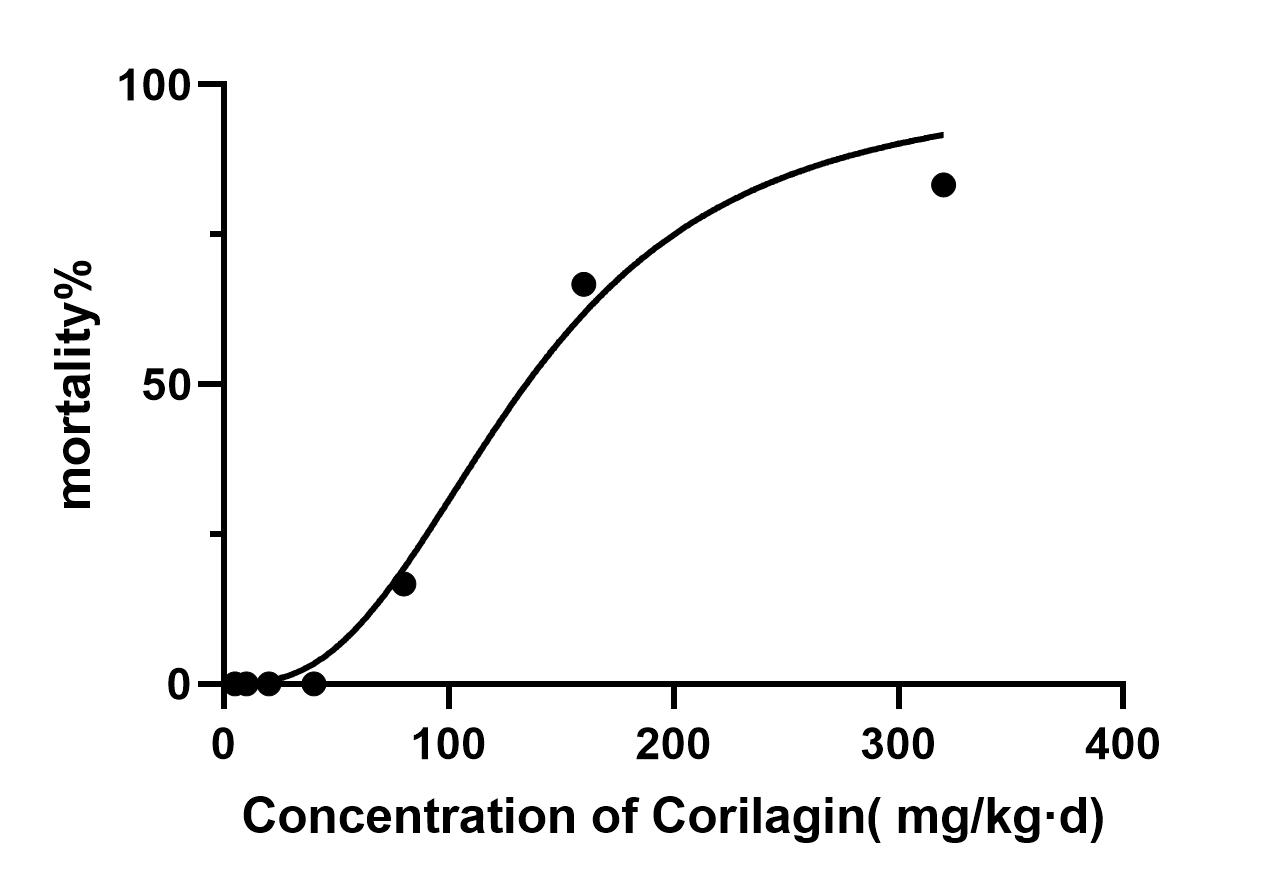

Supplement: Supplemental Figure 3 — Concentrations and mortality curve. We treated SD rats with corilagin from 5 to 160 mg/kg·d. No deaths occurred in 5, 10, 20, and 40 mg/kg·d groups (n = 6). One deaths occurred in 80 mg/kg·d group (n = 6), 4 deaths in 160 mg/kg·d group (n = 6), five deaths in 320 mg/kg·d group (n = 6). [file Image_3.JPEG]

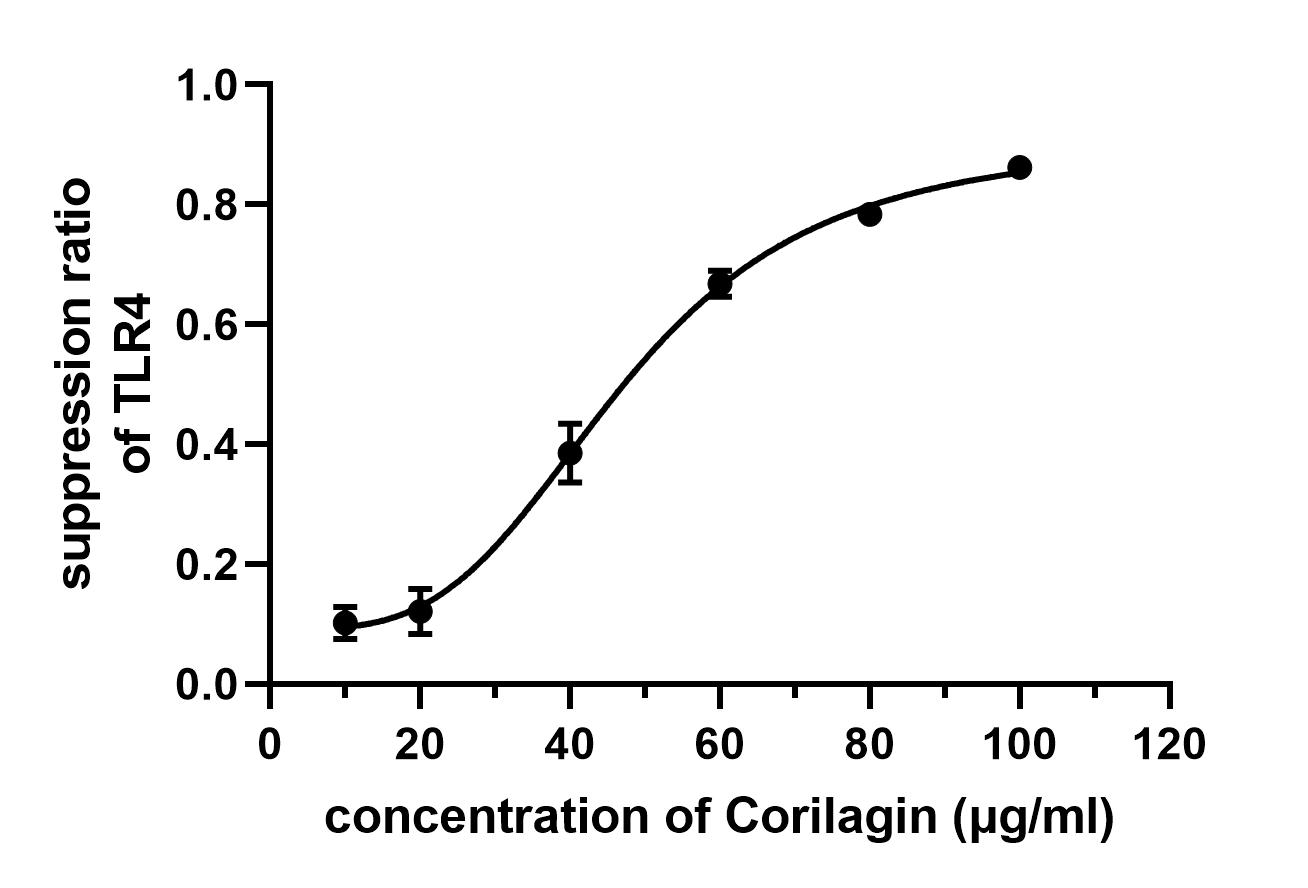

Supplement: Supplemental Figure 4 — Dose-dependent curve. Ana-1 cells were treated with 100, 80, 60, 40, 20, and 10 μg/ml corilagin after ox-LDL stimulation. mRNA expression of TLR4 was measured by qRT-PCR. Suppression ratio were calculated according to the results of qRT-PCR. The curve was “S”-shaped. [file Image_4.JPEG]

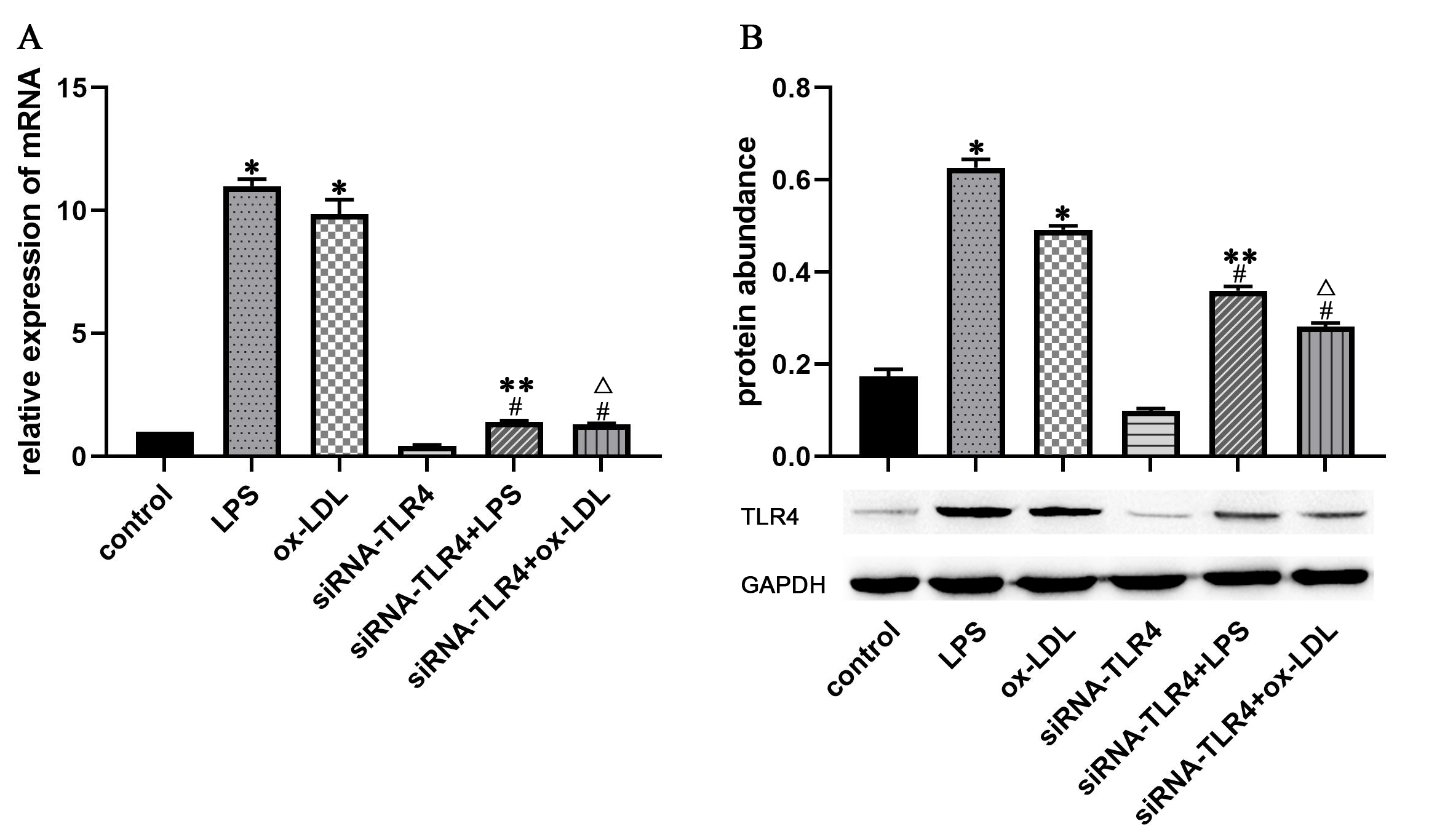

Supplement: Supplemental Figure 5 — Functional deficiency of TLR4 inhibition caused by siRNA. We stimulated the cells with LPS and ox-LDL after TLR4 downregulation. (A) mRNA expression of TLR4 was measured by qRT-PCR. *P < 0.05 compared with control group; #P < 0.05 compared with siRNA group; **P < 0.05 compared with LPS group; ΔP < 0.05 compared with ox-LDL group determined by one-way ANOVA method and subsequent S–N–K method (n = 5). (B) Protein expression was measured by Western blotting. *P < 0.05 compared with control group; #P < 0.05 compared with siRNA group; *P < 0.05 compared with LPS group; ΔP < 0.05 compared with ox-LDL group determined by one-way ANOVA method and subsequent S–N–K method (n = 5). [file Image_5.JPEG]

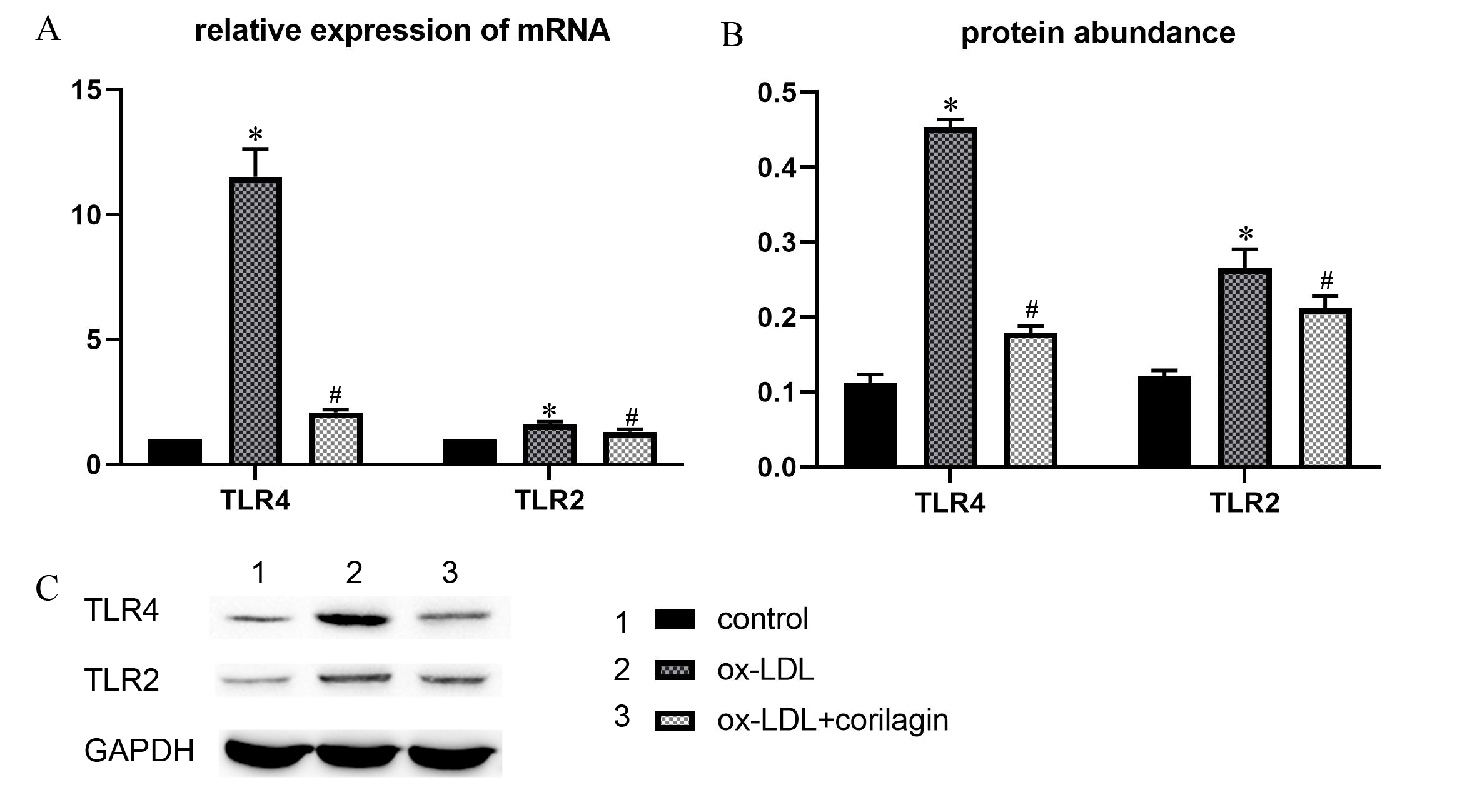

Supplement: Supplemental Figure 6 — Changes of TLR4 and TLR2 in atherosclerosis cell models. We treated Ana-1 cells with corilagin after ox-LDL stimulation. (A) mRNA expression was measured by qRT-PCR. *P < 0.05 compared with control group; #P < 0.05 compared with the model (ox-LDL) group determined by one-way ANOVA method and subsequent S–N–K method (n = 5). (B,C) Protein expression was measured by Western blotting. *P < 0.05 compared with control group; #P < 0.05 compared with the model (ox-LDL) group determined by one-way ANOVA method and subsequent S–N–K method (n = 5). [file Image_6.JPEG]
