# Supplementary material for: Corilagin Ameliorates Atherosclerosis in Peripheral Artery Disease via the Toll-Like Receptor-4 Signaling Pathway in vitro and in vivo
Source: Front Immunol. 2020 Aug 6;11:1611. doi: 10.3389/fimmu.2020.01611 (PMC7424006; doi:10.3389/fimmu.2020.01611)

unedited western blots in Figure 3

TLR4

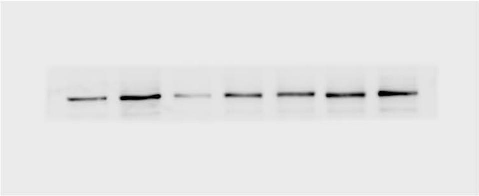

TIRAP

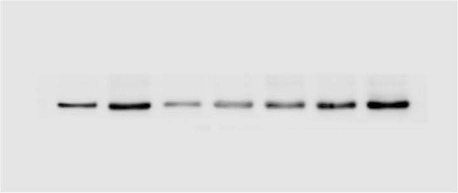

MyD88

TRAF6

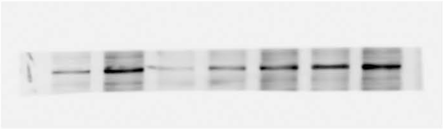

p38

NEMO

IRF5

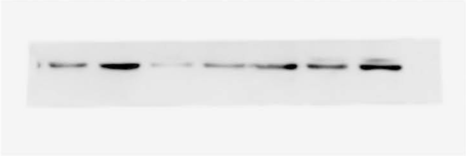

GAPDH

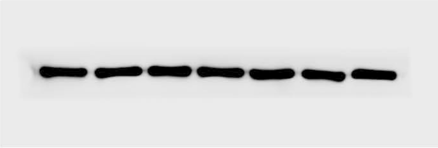

unedited western blots in Figure 4

TLR4

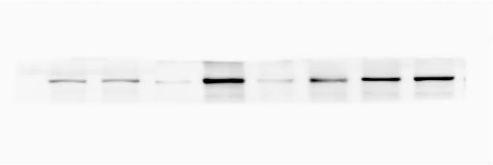

TIRAP

MyD88

TRAF6

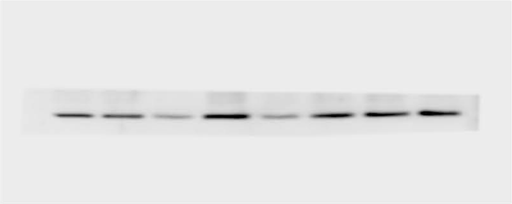

p38

NEMO

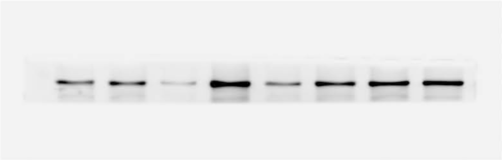

IRF5

GAPDH

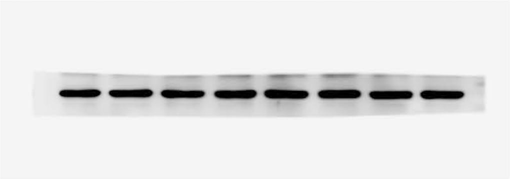

unedited western blots in Figure 5

TLR4

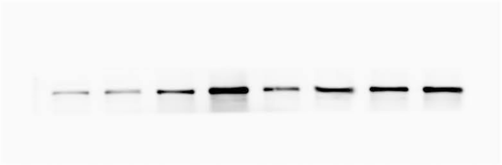

TIRAP

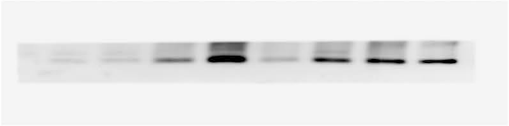

MyD88

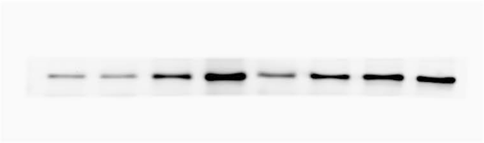

TRAF6

p38

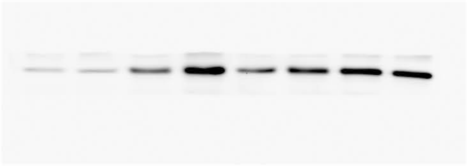

NEMO

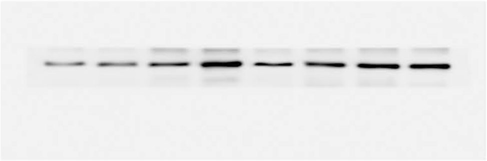

IRF5

GAPDH

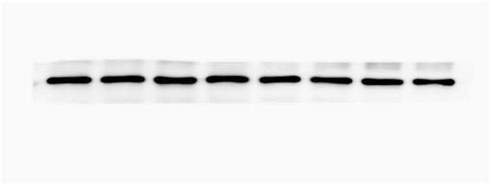

unedited western blots in Figure 6

TLR4

TIRAP

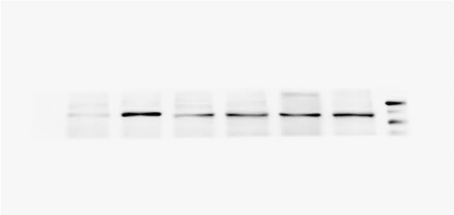

MyD88

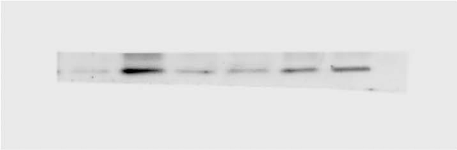

TRAF6

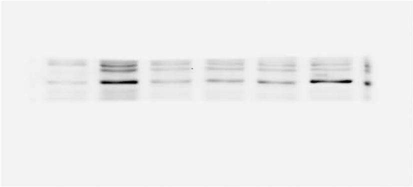

p38

NEMO

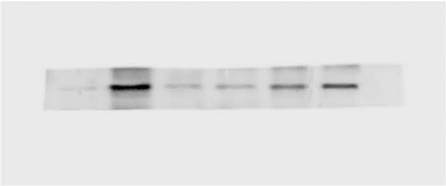

IRF5

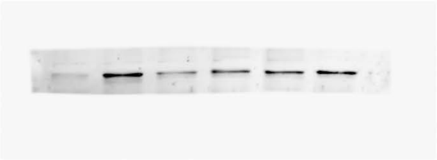

GAPDH

unedited western blots in Figure 7

TLR4

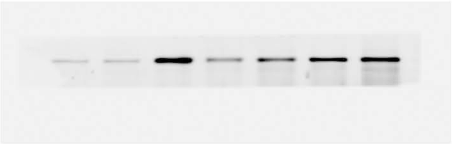

TIRAP

MyD88

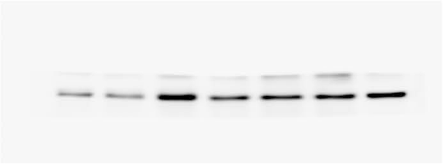

TRAF6

p38

NEMO

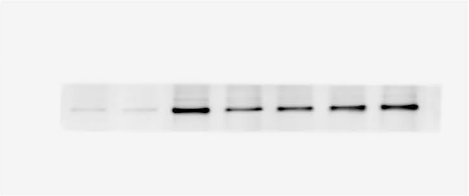

IRF5

GAPDH

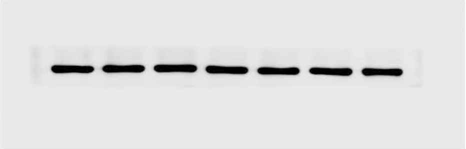

Supplement: Supplementary file 7 [file Image_7.PDF]
